# Delays and detours during cancer diagnosis: a cross-sectional study on patient pathways and provider intervals in public healthcare networks of Chile, Colombia and Ecuador

**DOI:** 10.1186/s12885-026-15737-5

**Published:** 2026-02-26

**Authors:** María-Luisa Vázquez, Pamela Eguiguren, Amparo-Susana Mogollón-Pérez, Andrés Peralta, Josep M Borràs, Ignacio Aznar-Lou, Signe Smith Jervelund, Carol Cardozo, Samar Benthami-Zarhouni, Iván Dueñas-Espín, María Luisa Garmendia, Sónia Dias, Ingrid Vargas

**Affiliations:** 1 Health Policy and Health Services Research Group, Health Policy Research Unit, Consortium for Health Care and Social Services of Catalonia, Barcelona, Spain; 2 Escuela de Salud Pública Dr. Salvador Allende Gossens, Facultad de Medicina, Universidad de Chile, Santiago de Chile, Chile; 3 Escuela de Medicina y Ciencias de la Salud, Universidad del Rosario, Bogotá, Colombia; 4 Grupo de Investigación en Servicios, Redes y Sistemas de Salud, Facultad de Salud y Bienestar, Instituto de Salud Pública Pontificia Universidad Católica del Ecuador, Quito, Ecuador; 5 University of Barcelona, Spain and Catalonian Cancer Plan, Department of Health, Spain; Bellvitge Biomedical Research Institute (IDIBELL), Barcelona, Spain; 6 Centro de Investigación Biomédica en Red de Epidemiología y Salud Pública (CIBERESP) Research and Development Unit, Institut de Recerca Sant Joan de Déu, Esplugues de Llobregat, Barcelona, Spain; 7 Department of Public Health, Faculty of Health and Medical Sciences, University of Copenhagen, Copenhagen, Denmark; 8 Grupo de Investigación en Inequidades y Determinantes Ecológicos y Ambientales en Salud (IDEAS), Instituto de Salud Pública, Facultad de Salud y Bienestar, Pontificia Universidad Católica del Ecuador, Quito, Ecuador; 9 Center for Research in Food Environments and Prevention of Nutrition-Related Chronic Diseases (CIAPEC), Institute of Nutrition and Food Technology, Universidad de Chile, Santiago, Chile; 10 NOVA National School of Public Health, Public Health Research Centre, Comprehensive Health Research Center (CHRC), REAL, CCAL, NOVA University Lisbon, Lisbon, Portugal

**Keywords:** Early diagnosis of cancer, Cancer diagnostic pathways, Provider intervals, Diagnostic delay, access to healthcare, Chile, Colombia, Ecuador

## Abstract

**Background:**

The longest delays in cancer diagnosis in Latin America occur between the first contact with health services and the confirmation of diagnosis (referred to as the provider interval), yet few studies have examined patient pathways to understand these delays. This study aims to analyze patient diagnostic pathways and their relationship to the provider interval in public healthcare networks of Chile, Colombia and Ecuador.

**Methods:**

Cross-sectional study based on questionnaire survey to adult patients diagnosed with cancer in prior 12 months in the study networks (*n* = 351 in Chile; 303 in Colombia; 365 in Ecuador). Study variables were diagnostic pathways (according to type and sequence of services) and provider intervals. Descriptive, bivariate and adjusted multivariate quantile regression analyses were conducted.

**Results:**

Two pathway types were identified: (a) public services use, frequent in Colombia (67.3%) and (b) mixed public-private services use, predominant in Chile (76.9%) and Ecuador (59.2%). Under 20% of patients followed the public pathway starting in primary care followed by referral to secondary care for confirmation. In Colombia and Ecuador, it was common to start in emergency departments, and in Colombia, going back and forth between care levels. Median provider interval was 111 days (IQR:134) in Chile, 156 (IQR:275) in Colombia and 92 (IQR:173) in Ecuador. Public-private pathways, more frequent in symptomatic patients, were significantly longer than public-only pathways (Chile: 116 vs. 92 days; Colombia: 175 vs. 153 days; Ecuador: 92 vs. 75 days; all *p* < 0.05). Emergency-initiated pathways were significantly shorter, whereas Colombia’s back-and-forth pathways were longer than those via primary care, although this difference was not statistically significant.

**Conclusion:**

The results reveal fragmented diagnostic pathways with significant delays, particularly in Colombia. The results underscore the need for equity-oriented policies to improve access to care in public healthcare networks.

**Supplementary Information:**

The online version contains supplementary material available at 10.1186/s12885-026-15737-5.

## Background

Delays in diagnosis and treatment contribute to the high and steadily increasing cancer mortality rates in Latin America [1]. Available studies [2–8] suggest that the longest delays occur in the *provider interval* (from first contact to services to diagnostic confirmation), and that are considerably longer than the *patient interval* (from symptoms discovery to first consultation) [9]. For patients diagnosed with breast cancer, for example, the median provider interval ranges from 60 to 195 days [2, 3, 6, 7, 10] compared to a median patient interval of 10 to 30 days [2, 3, 6, 7, 10]. This underscores critical delays within the healthcare system, which have not been observed in high-income countries (HICs) [11, 12].

There is no specific starting point or a single pathway to cancer diagnosis [13]. It can start through routes other than symptomatic presentation at health services such as screening programs or casual findings at any level of care. Furthermore, patients’ pathways may differ from that envisaged in primary care based health systems, which involves entry at primary care and referral to secondary or tertiary care for diagnostic confirmation [11, 12, 14]. Consequently, there is a growing interest in comparative analyses of diagnostic pathways to explain variations in provider intervals.

Available quantitative studies come mainly from HICs with health systems based on primary care, which acts as entry point to the services and coordinator of patient care throughout the care continuum [11, 12, 14]. In these, cancers are mostly identified by symptoms [11, 12], with presentation at primary care as the main route to diagnosis [11, 12, 15–17], and for certain cancers, also at emergency services [18]. Longer provider intervals were shown in patients whose cancer was symptom-detected [19], those referred from primary care [20] or that had used multiple specialized services [21]. However, these findings, cannot be extrapolated to fragmented health systems [22], such as those in Latin America, characterized by limited access, especially to secondary care, and poor cross-level coordination, where the patient journey to diagnostic confirmation might be highly complex [23]. In addition, research in Latin America is limited. Existing studies on provider intervals focused on breast cancer in Brazil [6, 24–26] and Mexico [2, 7, 8, 10], with few studies addressing other sites [2, 4, 5] or countries [3, 5, 27], and those on patient pathways considered only the initial and final contacts [25, 28]. The results showed long provider intervals, varying according to site, with primary care as the most frequent first contact, followed by private services [25, 28].

This study is part of a wider implementation research, EquityCancer-LA, aimed at evaluating the effectiveness of an integrated care intervention to improve early diagnosis of frequent cancers in Chile, Colombia and Ecuador [29]. It adopts a quasi-experimental design with an intervention and a control healthcare network in each country. The intervention impact on provider intervals and barriers to diagnosis is measured through a survey of patients diagnosed with cancer (pre and post). This study focuses on the public healthcare networks that provide care to the population insured by FONASA [National Health Fund] in Chile, those serving mainly the enrollees of the subsidized scheme of the Social Security System in Health and the uninsured in Colombia, and those of the Ministry of Health in Ecuador. In all three countries, the networks provide services to a defined population, with primary care as the entry point and coordinator of patient care along the care continuum [30]. The network providers are mostly public, except in Colombia, with a managed competition model, in which (mostly private) health insurers are responsible for organizing the healthcare networks for their enrollees, either through contracting with public or private providers or by providing services directly. In Chile, FONASA enrollees, except those in the lowest income group (A), can opt for the free choice modality and receive a voucher to use private services outside the public network, in addition to a co-payment.

All three countries have cancer control plans [31–34] focusing on early detection as the main strategy for improving cancer diagnosis and including opportunistic screening programs for prioritized cancer sites (breast and cervical in all three, and prostate and colorectal in Colombia and Ecuador). However, available studies show their limited implementation with low coverage rates and failures in providing timely diagnoses and treatments [1]. In addition, Chile and Colombia have promoted integrated care strategies including the establishment of maximum waiting times [33, 35, 36] and care pathways for prioritized cancer sites. Again, their impact on diagnostic delays might be limited, in Chile related to the defined criteria, and in Colombia, to its very limited implementation [37].

The aim of this study is to analyze patients’ diagnostic pathways from initial contact to services to confirmation and their relationship with the provider interval in public healthcare networks of Chile, Colombia and Ecuador. It presents quantitative results of the baseline study, which complement those of the qualitative study on factors influencing access to cancer diagnosis [37]. Both analyses have provided relevant information, first, to understand the complexity of access to cancer diagnosis and secondly, to guide the adaptation and scaling-up of tailored interventions to improve early diagnosis of cancer.

## Methods

### Design and study area

A cross-sectional study was conducted based on a survey using the EquityCancer-LA questionnaire to patients diagnosed with cancer in public healthcare networks of Chile, Colombia and Ecuador [29]. The study is grounded in a comprehensive conceptual framework for analyzing access to cancer diagnosis [29]. The model is built, on the one hand, on the model for the analysis of patient pathways developed by Walter et al. [13], which defines the intervals throughout the patient’s pathway from symptoms onset to diagnostic confirmation and treatment initiation, and, on the other hand, the access model by Aday and Andersen [38], which describes the factors related to population characteristics, health services, and health policies that influence and interact with each other throughout the pathway.

The study area in each country comprised two public healthcare networks, selected according to the following criteria: (a) provision of a continuum of services including at least primary care and secondary care /tertiary care, responsible for the diagnosis of cancer; (b) provision of services to a defined population; (c) provision of services mainly to areas with low to middle-low socioeconomic status. In Chile, the study area encompassed the northern and southern healthcare networks of Santiago; in Colombia, the health regions of the department of Cundinamarca; and in Ecuador the Southern (17D06) and Northern (17D03) health districts of Quito [29].

### Study population and sample

The study population comprised patients aged 18 years or more with a confirmed cancer diagnosis within the 12 months prior to the survey in the study networks and who resided in the study areas. Patients with consecutive cancers in same organ (recurrence) or synchronous primary cancers were excluded. In Chile and Ecuador, the most frequent cancer sites in the study area were selected: breast, colorectal, prostate, and stomach cancer, and lung cancer in Chile, and cervical cancer in Ecuador. In Chile, other cancer sites (urinary tract and testicular cancer) were also included as network managers were interested in reducing diagnostic delays. In Colombia, all cancer sites diagnosed within 18 months prior to the survey were selected (except hematological cancer). For the analysis of this study, forty-five patients in Colombia with non-melanoma skin cancer were excluded, as this cancer differs from the others in terms of clinical course and diagnostic characteristics (prolonged evolution, therapeutic management of some cases does not include histopathological confirmation).

The sample size was calculated considering the controlled before and after design of the study [39]. A sample size of 348 patients in each country (174 per network) was estimated to ensure the detection of a 15% variation in diagnostic intervals in a bilateral contrast (both between phases and between networks). As no previous empirical estimates were available, sample size was calculated assuming a worst-case scenario, a probability of 0.50 in the baseline/control network to maximize variance, and on the basis of a power of 80% (β = 0.20) and a confidence level of 95% (α = 0.05). Sample selection was adapted to the availability of cancer diagnosis records in each context. In Chile, a random, stratified proportional sampling was carried out based on the registry of confirmed cancer diagnoses (System for the Registration of Cancer Diagnoses Guaranteed by Law (SIGGES) for the period 2022–2023). Databases were cleaned by cross-referencing with mortality and health center databases, allowing the exclusion of deceased patients and misdiagnoses. In Colombia, consecutive sampling was conducted, recruiting patients identified from the databases of public providers and health authority, while in Ecuador, quota sampling was carried out based on the expected incidence of the selected cancers, recruiting patients both from the oncology service of the tertiary hospital and registries of the network’s hospitals. Random sampling was impossible in Ecuador and Colombia, due to the fragmentation and poor quality of the hospitals’ records, and in Colombia in addition, due to the refusal to participate of most insurers - that had to authorize access to their enrollees’ records - and the liquidation of the two that had agreed.

Patients were contacted for recruitment by telephone, and in Ecuador, also in person in oncology service, by the health services that treated them. In Colombia, and to a lesser extent in Ecuador, incorrect information in hospital records led to many unsuccessful contacts, including non-response or ineligible/deceased patients. A standardized protocol defined the number and timing of contact attempts. Non-responding, refusing, or deceased patients were replaced by the next on the list in Colombia and Ecuador, and randomly in Chile.

### Questionnaire

The EquityCancer-LA questionnaire was developed for this study to analyze time intervals, patient pathways influencing factors and barriers of access during cancer diagnosis (Additional File 1). The questionnaire is based on the study’s conceptual framework [29], the review of existing instruments [14, 40–42], available studies in Latin America [3, 6, 7, 10, 43] and the results of the qualitative study in the study areas [37]; from where the dimensions and variables were identified. In addition, the recommendations of the Aarhus statement [9] for the collection of data on key points in time and associated intervals during the cancer diagnosis were considered. The content of the questionnaire was validated through discussions with a multidisciplinary group of experts and adapted to contextual and language variants of each country. In each setting, a pre-test was conducted using face-to-face cognitive interviews with four patients diagnosed with cancer, followed by a pilot study on a sample of 20 to 27 patients, to evaluate interview pace, interviewer workload, acceptability, comprehensibility, and feasibility under real conditions. As a result, some questions were revised or removed to shorten or avoid repetition, and some adjustments were made to the order of the questions and the instructions for interviewers (https://equity-la.eu/instrumentos). The questionnaire is divided into 7 sections: (1) identification of the health problem and healthcare seeking; (2) patient’s pathways in the use of health services (public and private) from the first contact to diagnosis confirmation. The use of both public services (in Colombia, any health service contracted by the insurer for their enrollees), and private services through out-of-pocket expenses are accounted; (3–6) experience using the different care levels during the diagnosis (primary, outpatient secondary, emergency, and inpatient care); (7) co-morbidity and socio-demographic characteristics. The key points in time for calculating the different diagnostic intervals and access barriers from care seeking to diagnostic confirmation are contained in sections 1 to 6.

### Data collection and quality

Data were collected through personal interviews conducted by trained interviewers from August 2022 to December 2023 in Chile, to July 2024 in Ecuador, and to March 2025 in Colombia. The interviews were face-to-face in Chile and Ecuador, and by telephone in Ecuador and Colombia. Strategies to ensure the quality and consistency of data included careful training and close supervision of interviewers in the field or after each interview, review of all filled-in questionnaires, and re-contact interviewed patients to complete or clarify information. In addition, in Chile and Colombia they were validated against interview recordings, and in Colombia, 10% of randomly selected respondents were re-interviewed. Double-entry methods were used to control inconsistencies during data entry, except in Ecuador that used a digital questionnaire. In Chile and Ecuador information on the stage of the cancer was obtained from the records of the tertiary hospital in the network. In Colombia this information was not available due to the refusal of insurers to participate - that had to authorize access to their enrollees’ clinical records. Common rules for missing, incomplete, divergences on dates and ambiguous data were developed following other authors [12].

### Variables

The variables of study were patient pathways to cancer diagnosis and provider diagnostic intervals. The **patient pathways** were classified according to the type of service used - public (primary care, secondary outpatient care, emergency department and hospitalization) or private (medical visits, laboratory, emergency department, hospitalization) and the sequence of use from first contact to diagnostic confirmation. The number of contacts during the diagnostic process (visits, hospitalizations and tests) was also analyzed. **Provider interval** was defined following Aarhus statement [9] as the time elapsed between the date of the first appointment request for the symptoms (or realization of the opportunistic screening test/casual finding) and the diagnostic confirmation. For patients starting in primary care and being referred to secondary care, two subintervals were distinguished: primary care interval, or the time between the first visit to primary care and the referral to secondary care to continue with symptoms investigation for cancer suspicion, and secondary care interval or time between the date of referral from primary care to secondary care and the diagnostic confirmation.

Patients were classified according to **type of identification of the health problem**, as symptomatic, patients that sought health services because of the presences of symptoms, or non-symptomatic, those cases identified through opportunistic screening or casual findings during the investigation of another health problem.

### Data analysis

A descriptive analysis of patients’ pathways and intervals was carried out for each country. Time intervals (median, percentiles 75, 90 and interquartile range (IQR)) were stratified by type of identification of health problem, patient pathways and cancer site. Bivariate analyses were performed to identify any difference: (1) in the type of pathway according to the identification of the problem, using the chi-square test; and (2) (a) in the median provider interval in public pathways, between those entering through primary care and other public pathways, and (b) between the median primary and secondary care intervals, using the Mann-Whitney U test. P-values *<* 0.05 were considered statistically significant. Multivariate analyses were performed to identify any differences in the median provider interval (a) by type of identification and (b) by pathway, using quantile regression models adjusted by sex, age, and healthcare network, depending on the country. Statistically significant differences are shown in the tables. Sensitivity analyses were performed to assess the impact of outliers on model estimates. All analyses were performed using STATA (StataCorp. 2017. Stata Statistical Software: Release 15.0. College Station, TX: StataCorp LLC.)

## Results

### Sample characteristics and participation

Of the 8,317 patients contacted, only 6,013 (72.32%) responded. Among those, 1,510 fulfilled the inclusion criteria and were alive (70% in Chile, 16% in Colombia, and 31% in Ecuador), of which, 1,064 patients participated (70.5%). For the analysis of this study, forty-five patients in Colombia with non-melanoma skin cancer were excluded, thus finally, 1,019 (97.3%) patients were included in the analysis. The patient flow with patient identification, responses and rejections for each country is depicted in Table 1S.

In the samples from the three countries, most patients were women (varying according to the cancer site) with a predominance of the older age group (60 to 79 years), except in Ecuador, where the predominant group was younger (40 to 59 years). In Colombia, 70.6% had no education or only primary education, while in Chile it was 48.2% and in Ecuador, 37.2%. In Chile, most participants were affiliated to categories B (55.8%) and C/D (30.2%) of FONASA, which correspond to higher income levels, while in Colombia mostly to the subsidized system (72.6%), and in Ecuador covered by the Health Ministry (95.3%). In all three countries, breast and colorectal were the most frequent cancer sites, along with cervix and stomach in Ecuador and Colombia, and prostate in Chile. In Ecuador, 45.2% of patients had advanced stages (III and IV) at diagnosis, and 38.5% in Chile. Most participants had identified the health problem by the presence of symptoms, although in Chile the percentage of those detected by casual finding/opportunistic screening was high (40.5%) (Table 1).

**Table 1:** Characteristics of the sample in the study networks of Chile, Colombia, and Ecuador

Variables	Chile *N* = 351	Colombia *N* = 303	Ecuador *N* = 365
	*n* (%)	*n* (%)	*n* (%)
Sex			
Woman	206 (58.7)	198 (65.4)	304 (83.3)
Man	145 (41.3)	105 (34.6)	59 (16.2)
Age			
18–39	20 (5.7)	29 (9.6)	50 (13.7)
40–59	109 (31.1)	107 (35.3)	161 (44.1)
60–79	204 (58.1)	139 (45.9)	125 (34.3)
80 or more	18 (5.1)	28 (9.2)	13 (3.6)
Level of education			
Non/incomplete primary	88 (25.1)	111 (36.6)	53 (14.5)
Primary	81 (23.1)	103 (34.0)	83 (22.7)
Secondary and further	182 (51.9)	84 (27.7)	225 (61.6)
Type of public insurance			
Fonasa A	25 (7.1)		
Fonasa B	196 (55.8)		
Fonasa C and D	106 (30.2)		
Subsidized		220 (72.6)	
Contributory and Special		83 (27.4)	
MSP			342 (95.3)
IESS ISSPOL, ISSFA			17 (4.7)
Cancer site			
Breast	131 (37.3)	51 (16.8)	177 (48.5)
Colorectal	89 (25.4)	49 (16.2)	54 (14.8)
Prostate	61 (17.4)	27 (8.9)	28 (7.7)
Lung	23 (6.6)	7 (2.3)	*NA*
Urinary tract (kidney and bladder)	21 (6.0)	14 (4.6)	*NA*
Stomach	19 (5.4)	41 (13.5)	43 (11.8)
Cervix	*NA*	53 (17.5)	63 (17.3)
Testicle	7 (2.0)	3 (1.0)	*NA*
Melanoma	*NA*	3 (1.0)	*NA*
Other	*NA*	55 (18.2)	*NA*
Cancer stage			
I	110 (31.3)	*n/a*	22 (6.0)
II	99 (28.2)	*n/a*	99 (27.1)
III	80 (22.8)	*n/a*	107 (29.3)
IV	55 (15.7)	*n/a*	58 (15.9)
* Missing*	*7 (2.0)*	*n/a*	*79 (21.6)*
Identification of the health problem			
Symptomatic	209 (59.5)	262 (86.5)	308 (84.4)
Non symptomatic	142 (40.5)	41 (13.5)	57 (15.6)

*MSP* Ministry of Public Health, *IESS* Ecuadorian Institute of Social Security, *ISSPOL* Social Security Institute of the National Police, *ISSFA* Social Security Institute of the Armed Forces

Category other type of cancer in Colombia: thyroid, ovary, brain, vagina, pancreas, anal, soft tissue, nasopharyngeal, tongue, penis, larynx, eye, salivary/parotid, cerebellum, liver, esophagus, appendix, vesicle.

*NA* Not applicable, as selected cancer were different among countries; n/a: Data on cancer stage was not available for Colombia

Data source of cancer stage: Clinical registers in Chile and Ecuador

### Patient’s pathways until cancer diagnosis

Although the diagnostic pathways varied across countries, two main types were identified: those who used only public services until the diagnosis was confirmed, frequent in Colombia (67.3%), and those who also used private services, frequent in Chile (76.9%) and in Ecuador (59.2%) (Table 2).

**Table 2:** Patient’s pathways until cancer diagnosis in Chile, Colombia, and Ecuador

	Chile *N* = 351	Colombia *N* = 303	Ecuador *N* = 365
	*n*(%)^1^	*n*(%)^1^	*n*(%)^1^
**Diagnostic pathways only in public services**	81 (23.1)	204 (67.3)	149 (40.8)
Entry by primary care, referred to secondary care(OC) and diagnosed after few visits in secondary care (< 3) services	56 (16.0)	54 (17.8)	67 (18.4)
Entry by ES and use only of specialized services until diagnosis	13 (3.7)	60 (19.8)	40 (11.0)
Entry by secondary care(OC) and diagnosed after use of secondary care services (OC, hospitalization, ES)	6 (1.7)	20 (6.6)	30 (8.2)
Other pathways involving going back and forth between services and/or levels of care	6 (1.7)	70 (23.1)	12 (3.3)
Entry by primary care and use mostly of ES and hospitalization	2 (0.6)	23 (7.6)	3 (0.8)
Entry by primary care/secondary care and back and forth between care levels	4 (1.1)	27 (8.9)	5 (1.4)
Entry by primary care, after few visits (primary care ≤ 3) referred to secondary care(OC), and use of different specialized services (OC, hospitalization, ES)	0 (0.0)	20 (6.6)	4 (1.1)
**Diagnostic pathways in public and private services**	270 (76.9)	99 (32.7)	216 (59.2)
Public pathways complementing with some private services (diagnostic tests, consultations and hospitalizations)	108 (30.8)	92 (30.4)	192 (52.6)
Private pathways ending in public services for diagnostic confirmation and referral to treatment	162 (46.2)	7 (2.3)	24 (6.6)

^1^Percentages calculated over total number of patients in each country

(OC): Secondary care outpatient consultation; ES Emergency Services

For Ecuador, health services of the complementary health network (IESS, ISSFA o ISSPOL) are included in the public healthcare services categories

Regarding the public pathways, in all three countries less than 20% of patients had the first contact in primary care followed by a referral to secondary care for diagnostic confirmation. In Colombia, the most frequent pathways were of patients who went back and forth between levels of care and/or specialized services (emergency room, outpatient visits and hospitalization) (23.1%), and of those who entered through the emergency services and had subsequent use of various specialized services until confirmation (19.8%). The back-and-forth pathway between care levels was very infrequent in Chile and Ecuador. In Ecuador, pathways starting in emergency services (11.0%) and in outpatient secondary care (8.2%) were also frequent (Table 2).

Regarding public-private pathways, those that predominantly used public services complemented with some private services for diagnostic tests and/or consultations were frequent (Chile: 30.8%; Colombia: 30.4% and Ecuador.: 52.6%), although Chile also had a high percentage of patients who mainly utilized private services and ended up in public services for diagnostic confirmation and referral to treatment (46.2%) (Table 2).

The pathways varied according to the type of identification of the health problem; public-private pathways were significantly more frequent among symptomatic patients in Chile and Colombia (Chile:83.3%, Colombia: 35.1%; *p* < 0.01 in both cases) (Table 4S).

The number of health services contacts varied according to the type of pathway. Those with a high median number of contacts were public-private in all three countries (Chile: 7 contacts (IQR: 6–9); Colombia: 7 contacts (IQR:5–9); Ecuador: 4 contacts (IQR:3–5)), and public going back and forth between services, particularly in Colombia (7 contacts (IQR:5–8)) (Table 5 S).

### Provider interval to cancer diagnosis

#### All patients and cancer sites

The median provider interval for all patients ranged from 92 (IQR: 173) days in Ecuador, 111 (IQR: 134) in Chile, to 156 (IQR: 275) in Colombia (Table 3). At the 75 percentile it varied between 6.5 months in Chile and 11 months in Colombia. 21% of patients in Colombia had a provider interval exceeding 365 days, whereas the respective proportions in Chile and Ecuador were 12% and 14% respectively (Table 6 S).

**Table 3:** Provider intervals (in days) to cancer diagnosis for all patients and by symptomatic and non-symptomatic

	Chile	Colombia	Ecuador
**All patients**	(*n* = 351)	(*n* = 303)	(*n* = 365)
Provider interval	(*n* = 350)	(*n* = 292)	(*n* = 365)
Percentile 50	111	156	92
Percentile 75	195	339	212
Percentile 90	426	670	454
IQR	134	275	173
* Missing*	*1*	*11*	8
Primary care interval	(*n* = 66)*	(*n* = 143)	(*n* = 166)
Percentile 50	15^1^	21^1^	10^1^
Percentile 75	41	174	46
Percentile 90	111	407	119
IQR	42	174	46
* Missing*	*0*	*9*	46
Secondary care interval (of patients referred to secondary care)	(*n* = 267)	(*n* = 135)	(*n* = 166)
Percentile 50	61^1^	143^1^	62^1^
Percentile 75	116	212	150
Percentile 90	202	340	328
IQR	86	145	121
* Missing*	*0*	*7*	42
**Symptomatic patients**	(*n* = 209)	(*n* = 262)	(*n* = 308)
Provider interval	(*n* = 208)	(*n* = 255)	(*n* = 300)
Percentile 50	111	154	92
Percentile 75	206	334	212
Percentile 90	461	674	454
IQR	149	273	174
* Missing*	*1*	*7*	8
Primary care interval	(*n* = 47)*	(*n* = 126)	(*n* = 173)
Percentile 50	16	31	10
Percentile 75	42	182	45
Percentile 90	196	511	111
IQR	43	182	45
* Missing*	*0*	*7*	*74*
Secondary care interval (of patients referred to secondary care)	(*n* = 161)	(*n* = 118)	(*n* = 136)
Percentile 50	55	113	62
Percentile 75	103	211	143
Percentile 90	156	340	304
IQR	77	147	114
* Missing*	0	*5*	*33*
**Non-symptomatic patients**	(*n* = 142)	(*n* = 41)	(*n* = 57)
Provider interval	(*n* = 142)	(*n* = 37)	(*n* = 57)
Percentile 50	114	191	92
Percentile 75	191	365	245
Percentile 90	351	625	487
IQR	130	268	200
* Missing*	*0*	*4*	*0*
Primary care interval	(*n* = 19)*	(*n* = 17)	(*n* = 32)
Percentile 50	14	0	20
Percentile 75	41	19	61
Percentile 90	72	366	212
IQR	41	19	61
* Missing*	*0*	*2*	*11*
Secondary care interval (of patients referred to secondary care)	(*n* = 106)	(*n* = 17)	(*n* = 30)
Percentile 50	76	144	70
Percentile 75	143	244	227
Percentile 90	222	320	446
IQR	109	175	186
* Missing*	0	*2*	9

Provider interval: Time from appointment request to the date of diagnosis confirmation. Primary care interval: Time from first consultation in primary care to referral to secondary care (OC). Secondary care interval: Time from referral to secondary care from primary care to diagnosis confirmation. *It only includes patients with public pathways or public complementing with some private services who have been referred by primary care doctors

^1^Statistically significant differences between the median primary care interval and the median secondary care interval in all patients, using Mann-Whitney U test (*p* < 0.001))

The median secondary care interval ranged from 61 days in Chile, 62 Ecuador to 143 days in Colombia and was longer than the primary care interval (Chile: 15, IQR: 42; Colombia: 21 IQR 174; Ecuador: 10, IQR 46; all *p* < 0.001) (Table 3). There were differences in the median provider interval according to the most frequent cancer sites in each country. In Chile, the interval appeared to be longest for prostate cancer (median of 224 days) and in Colombia and Ecuador, for breast cancer (medians of 212 and 104 days, respectively) (Table 3S).

#### According to type of identification of the health problem

The median provider interval for non-symptomatic patients was similar to that for symptomatic patients in the three countries (Chile:114, IQR: 130; Colombia: 191, IQR: 268; Ecuador 92, IQR: 200; all *p* > 0.05) (Tables 2 and 3S).

#### According to type of pathway

Differences in the provider interval according to the patients’ pathway were observed within each country. In all three countries, the median of those that used public and private services during diagnosis was significantly longer than those that only used public services (Chile: 116 vs. 92 days; Colombia: 175 vs. 153; Ecuador: 92 vs. 75, all *p* < 0.05) (Table 4). The median for those who used mainly private services and public services only for confirmation and referral to treatment appeared to be longer than public-only in Chile (111 days), and shorter in Colombia (94 days) and Ecuador (51 days).

**Table 4:** Provider interval (in days) according to type of patient pathway to cancer diagnosis

	Chile	Colombia	Ecuador
	(*n* = 351)	(*n* = 303)	(*n* = 365)
**Diagnostic pathways only in public services**			
All patients	*n* = 81	*n* = 195	*n* = 149
Percentile 50	92^1^	153^1^	75^1^
Percentile 75	148	327	200
Percentile 90	291	611	396
IQR	86	266	169
* Missing*	0	9	2
Entry by primary care, referred to secondary care(OC) and diagnosed after few visits in secondary care (< 3) services	*n* = 56	*n* = 51	*n* = 67
Percentile 50	102^2^	192^2^	135^2^
Percentile 75	174	371	273
Percentile 90	425	495	780
IQR	111	259	212
* Missing*	0	3	1
Entry by ES and use only of specialized services until diagnosis	*n* = 13	*n* = 59	*n* = 40
Percentile 50	29^2^	49^2^	30^2^
Percentile 75	47	92	92
Percentile 90	92	170	227
IQR	49	76	82
* Missing*	0	1	1
Entry by secondary care(OC) and diagnosed after use of secondary care services (OC, hospitalization, ES)	*n* = 6	*n* = 18	*n* = 30
Percentile 50	151	102	60^2^
Percentile 75	184	242	144
Percentile 90	258	691	317
IQR	88	181	113
* Missing*	0	2	0
Other pathways involving going back and forth between services and/or levels of care	*n* = 6	*n* = 67	*n* = 12
Percentile 50	43	293	172
Percentile 75	114	446	382
Percentile 90	291	999	474
IQR	146	331	291
* Missing*	0	3	0
**Diagnostic pathways in public and private services**			
All patients	*n* = 269	*n* = 97	*n* = 216
Percentile 50	116^1^	175^1^	92^1^
Percentile 75	213	349	235
Percentile 90	446	812	459
IQR	158	261	188
* Missing*	1	2	6
Public pathways complementing with some private services	*n* = 107	*n* = 91	*n* = 192
Percentile 50	145	183	108
Percentile 75	298	370	242
Percentile 90	702	812	467
IQR	233	278	183
* Missing*	1	1	5
Private pathways ending in public services	*n* = 162	*n* = 6	*n* = 24
Percentile 50	111	94	51
Percentile 75	169	112	92
Percentile 90	306	328	270
IQR	109	53	62
* Missing*	0	1	1

Secondary care (OC): Secondary public care outpatient consultation; ES Emergency Services

For Ecuador, health services of the complementary health network (IESS, ISSFA o ISSPOL) are included in the public healthcare services categories

^1^Statistically significant differences when comparing the median provider interval with the median of diagnostic pathways in public and private services, using a quantile regression model adjusted by sex, age and healthcare network (*p* < 0.05)

^2^Statistically significant differences when comparing the median provider interval with the median of pathways starting in primary care followed by a referral to secondary care for diagnostic confirmation using Mann-Whitney U test (*p* < 0.01)

Regarding the public pathways, the median provider interval of those starting in primary care and then referred to secondary care varied between 102 days in Chile and 192 in Colombia. The pathways with significantly shorter median times than the pathway entering through primary care in all three countries were those starting in the emergency room, ranging from 29 days in Chile to 49 days in Colombia (*p* < 0.01 in both cases). In Ecuador, those patients who started directly in outpatient secondary care services also had significantly shorter provider interval (60 days, *p* = 0.01). In Colombia, the pathways involving going back and forth between levels of care and/or services had a higher provider interval median of 293 days (15 months at the 75th percentile); however, this difference was not statistically significant (*p* = 0.311).

## Discussion

To the best of our knowledge, this study represents the first attempt to explore and compare patient pathways and their relationship with provider intervals in different health systems in Latin American countries. One of its main strengths is the novel use of a patient survey applying a common questionnaire to comprehensively analyze the patient’s pathway and capture diagnostic time points along the pathway. This study adds to a growing body of evidence on diagnostic pathways and time intervals during cancer diagnoses [11, 14], providing specific information for highly fragmented healthcare systems.

The results reveal the frequency of fragmented diagnostic pathways: on the one hand, pathways involving the use of public and private services, predominant in Chile and, to a lesser extent, in Ecuador; and on the other hand, pathways involving repeated visits to services and/or to different levels of care, which are frequent in Colombia. The study also identifies long intervals between the first contact to health services and the confirmation of the cancer diagnosis in all three countries, but especially in Colombia, with even longer intervals for fragmented diagnostic pathways and for certain cancer sites. However, no differences were found according to the type of problem identification (symptomatic/non symptomatic). It is noteworthy that the median provider interval is similar in Chile and Ecuador, even though, on the one hand, income levels and per capita public health expenditure are significantly higher in Chile [44] and, on the other hand, the percentage of patients in early stages at diagnosis, as well as asymptomatic patients, is higher in the Chilean sample. These results could be related to a high prevalence of public-private pathways with long provider intervals in Chile and, conversely, in Ecuador, of pathways beginning in emergency departments or outpatient secondary care consultations, with short intervals.

The median provider interval, ranging from 3 months in Ecuador to 5 months in Colombia exceeds international recommendations (between two and four weeks) [45], which may have an impact on the stage of the disease at the time of diagnosis and, consequently, on prognosis [46]. Moreover, it should be taken into account that sample comprises only confirmed cancer patients who are alive; consequently, diagnostic delays are likely to be longer in the overall patient population. Delays are notable for certain cancer sites: breast and cervical cancer in Colombia, and to a lesser extent in Ecuador, and prostate cancer in Chile. Provider interval are far from those reported in HICs for breast cancer (from 13 to 29 days) [11], cervical cancer (67 days) [47], and prostate cancer (from 57 to 112 days) [48]. It is striking that the provider interval for breast and cervical cancer in Colombia and Ecuador is longer than for other cancers (e.g. colorectal cancer), as these are usually among the cancers with the shortest intervals [47, 49]. The median provider interval in Colombia for both cancers (212 and 183 days, respectively) is even longer than that reported by existing studies in Latin America for breast cancer (ranging from 53 to 195 days) [7, 28] and cervical cancer (99 days) [2], as well as by other studies in Colombia for breast cancer (53 to 91 days) [3, 27]. The difference with other national studies might be attributed to the predominance of patients enrolled in the subsidized regime, with poorer healthcare access. In the case of prostate cancer, although it is usually among the cancers with the longest provider intervals [49], the median reported for Chile (224 days) is even higher than the only existing study in Latin America (107 days) [2].

Contrary to expectations, the provider interval for non-symptomatic patients diagnosed during the investigation of another health problem (opportunistic screening/casual finding) is similar to that of patients who are diagnosed based on symptoms. The results also show longer secondary care intervals for asymptomatic patients, indicating that difficulties are concentrated mainly after referral from primary care. This may be related to deficits in the implementation of rapid referral pathways in general and particularly in screening programs [1].

Although all three health systems are based on primary care models, the expected pathway of entry at primary care and referral to secondary care for further investigation and diagnostic confirmation is not the most frequent and its median provider interval is long, particularly in Colombia. However, in contrast to studies in HICs, the greatest delay does not occur during the assessment in primary care, but rather from referral to diagnostic confirmation at the secondary level. This reveals the urgent need to implement fast-track referral pathways for patients with agreed warning signs and symptoms that lead to a suspicion of cancer.

Instead, fragmented patient trajectories are more prevalent, involving back-and-forth visits between public and private services and/or levels of care, in which waiting times for the different services used accumulate the time to diagnosis [37]. The causes of diagnostic delays could be related to the confluence of numerous barriers of access to cancer diagnosis identified in the qualitative study from the institutional stakeholders perspective [37], in line with other qualitative studies in the region [43, 50–53]. Some are common to all three countries, such as structural barriers (deficits in diagnostic resources and geographical accessibility of healthcare networks) which lead to long waiting times, especially after referral, as well as shortfalls in primary care doctors’ knowledge, resulting in delayed patient referral. But there are also country specific barriers: in Chile, prioritization health policy that increases delays for non-prioritized conditions; in Ecuador, underfunding of the public healthcare system; and in Colombia, the management of care by insurers. According to the results of the qualitative study [37], the longer provider intervals in Colombia might be explained by the use of managed care mechanisms, such as authorizations for tests and consultations, which prolong waiting times and often lead to the rejection of tests requested by primary care doctors, hindering diagnosis, or the fragmented purchase of services (tests, consultations) during the diagnostic episode, or the short duration of contracts with providers, which forces patients to visit multiple providers and sometimes restart the referral process.

One of the most relevant findings of this study is that the median interval of the public-private pathway is significantly longer than that of public-only pathways (Chile: 116 vs. 92 days; Colombia: 175 vs. 153; Ecuador: 92 vs. 75, all *p* < 0.05), challenging the general belief that the use of private services to circumvent long waiting times inpublic services speeds up the diagnostic process [37, 53]. International evidence on the influence of private use on cancer diagnostic delay is scarce and contradictory, probably due to a partial analysis focused only on the influence of certain elements such as the type of entry into services (public or private).

The use of private services during cancer diagnosis could also entail high out-of-pocket expenses incurred by patients, impacting on equity in access to cancer diagnosis and, therefore, on the start of treatment and survival. Although further research is needed, the lower proportion of beneficiaries with the lowest income level (FONASA A) in the randomized sample in Chile, compared to the population residing in the area [54], could indicate a higher probability of being diagnosed, and subsequently treated, by those patients with the ability to pay for private testing.

Finally, the results also show alternative trajectories to those expected but with shorter provider intervals, such as those of patients admitted through the emergency department, which are more frequent in Colombia and Ecuador. These types of pathways, which are often associated with a worse patient experience [55] and outcome [18], reflect existing barriers of access, as these may be patients admitted at advanced stages of cancer with complications in their clinical picture, or patients who, in an attempt to overcome existing barriers, seek urgent care to speed up the diagnostic process. In Ecuador, there are also shorter intervals for patients who are admitted directly through secondary care and those who follow a predominantly private pathway and end up in public services to start treatment. The results of the qualitative study [37] show that the first type of pathway is probably facilitated by informal relationships with healthcare professionals, and reflects the inequalities in access to the public health system generated by the two types of pathways.

This study has some limitations. The findings cannot be generalized to the countries as a whole, since the study was conducted in specific public healthcare networks. However, the study provides valuable evidence on critical aspects for early cancer diagnosis policy design in contexts in which information is scarce. Recall bias could have affected the accuracy of the measurement of intervals based on dates provided by patients. To minimize this, calendar aids were used to recall dates during interviews. The selection of patients up to 12–18 months after diagnosis may have led to the exclusion of more advanced cases with longer intervals, due to high mortality, potentially underestimating the provider interval.

Further research is needed to expand the findings of this study including analytical studies of associations such as private services use and diagnostic delays in public healthcare networks, taking into account the full diagnostic pathway, as well as population-based evaluations of provider intervals that include the time intervals of non-survivors, and have adequate sample sizes to analyze social inequalities in access to early cancer diagnosis, which will require public investment in the creation of these registries.

## Conclusion

This study provides the first comprehensive assessment of diagnostic pathways and their relation to provider intervals in different Latin American health systems. The findings reveal fragmented patient pathways, and substantial delays in diagnosis of most frequent and prioritized cancers in the three countries (breast, cervical, prostate and colorectal cancer), underscoring the need for policies that address the structural and organizational barriers driving these delays in order to strengthen public healthcare networks and improve equity of access to early diagnosis of cancer. The use of private services during diagnosis may reinforce inequities in access and, as shown, does not appear to substantially reduce delays. Other unexpected findings—including longer diagnostic intervals in secondary care, similar delays among symptomatic and asymptomatic patients, and extended intervals in prioritized cancers—point to additional critical gaps that require targeted interventions. These include, on the one hand, the implementation of multicomponent strategies combining training of primary care doctors in diagnostic suspicion, the establishment of clinical agreement and fast-track referral pathways between primary care and secondary/tertiary care for patients with cancer suspicion which, to be effective, must be adapted to the local context and to the priority cancers in each country. On the other hand, they include reviewing the diagnostic circuits in existing screening programs.

## Supplementary Information

Additional file 1. EquityCancer-LA Questionnaire (English version)

Additional file 2. Table 1S. Flow of patient identification, responses and rejection in Chile, Colombia and Ecuador. Table 2S. Diagnostic intervals (in days) to cancer diagnosis for all patients, and according to symptomatic and non-symptomatic in the studied public healthcare networks of Chile, Colombia, and Ecuador. Table 3S. Diagnostic intervals (in days) to cancer diagnosis according to most frequent cancer site in the studied public healthcare networks of Chile, Colombia, and Ecuador. Table 4S. Patient pathways to cancer diagnosis for symptomatic and non-symptomatic patients according to type of pathway (public or private) in Chile, Colombia, and Ecuador. Table 5S. Number of contacts to health services (visits, hospitalizations and tests) until cancer diagnosis according to patient pathways in Chile, Colombia and Ecuador. Table 6S. Characteristics of patients with a provider interval longer than 365 days in the studied networks of Chile, Colombia and Ecuador.in Latin America.

## Data Availability

The data supporting this study’s findings will not be available in a public repository until the project is finalized. However, they may be received from the corresponding author upon reasonable request.

## Declarations

Conditions of study procedure, risk and benefit assessment, confidentiality, privacy, and informed consent were all approved by the corresponding ethical committees in the participating countries [29]. In addition, confidentiality agreements were signed with all participating institutions. Written informed consent was obtained from every interviewee, after being informed that participation was voluntary and that they were free to decline or withdraw without any negative repercussions. Data were coded and processed in such a way that the individual cannot be identified and appropriately stored.

## Abbreviations

HIC: High–Income Countries
WHO: World Health Organization
IQR: Interquartile Range

## References

[1] Goss PE, Lee BL, Badovinac-Crnjevic T, Strasser-Weippl K, Chavarri-Guerra Y, St Louis J et al. Planning cancer control in Latin America and the Caribbean. Lancet Oncol. 2013;14(5):391–436. Available from: http://www.ncbi.nlm.nih.gov/pubmed/23628188 10.1016/S1470-2045(13)70048-2 23628188

[2] Unger-Saldaña K, Arroyo-Valerio A, Turrubiates GS, Gómez-Navarro JA, Bargalló-Rocha E, Quintero-Beuló G et al. Time intervals to care and health service use experiences of uninsured cancer patients treated under public financing in Mexico City. Cancer Epidemiol. 2023;84(February):102366. Available from: http://www.ncbi.nlm.nih.gov/pubmed/37086645 10.1016/j.canep.2023.102366 37086645

[3] Piñeros M, Sánchez R, Perry F, García OA, Ocampo R, Cendales R. [Delay for diagnosis and treatment of breast cancer in Bogotá, Colombia]. Salud Publica Mex. 2011;53(6):478–85. Available from: http://www.ncbi.nlm.nih.gov/pubmed/22282140 22282140

[4] Souza RHS, Maluf EMCP, Sartor MC, de Carvalho DS. Colorectal cancer: factors related to late diagnosis in users of the public health system treated at an Universitary Hospital in Curitiba, Paraná State, Brazil. Arq Gastroenterol. 2016;53(2):68–75. Available from: http://www.scielo.br/scielo.php?script=sci_arttext&pid=S0004-28032016000200068&lng=en&tlng=en 10.1590/S0004-28032016000200004 27305411

[5] Valdés Díaz S, García Silvera E, Pérez Cruz H, Hernández Hernández M. Length of diagnostic delay in patients with non-small-cell lung cancer. MEDICC Rev. 2010;12(1):29–32. 20387332 10.37757/MR2010.V12.N1.6

[6] Rezende MCR, Koch HA, Figueiredo J, de Thuler A. Causas do retardo na confirmação diagnóstica de lesões mamárias em mulheres atendidas em um centro de referência do Sistema Único de Saúde no Rio de Janeiro. Rev Bras Ginecol e Obstet. 2009;31(2):75–81. 10.1590/s0100-72032009000200005 19407912

[7] Unger-Saldaña K, Miranda A, Zarco-Espinosa G, Mainero-Ratchelous F, Bargallõ-Rocha E. Miguel Lázaro-Leõn J. Health system delay and its effect on clinical stage of breast cancer: Multicenter study. Cancer. 2015;121(13):2198–206. 25809536 10.1002/cncr.29331 PMC6681165

[8] Bright K, Barghash M, Donach M, de la Barrera MG, Schneider RJ, Formenti SC. The role of health system factors in delaying final diagnosis and treatment of breast cancer in Mexico City. Mexico Breast. 2011;20(Suppl 2):S54–9. 21371885 10.1016/j.breast.2011.02.012

[9] Weller D, Vedsted P, Rubin G, Walter FM, Emery J, Scott S et al. The Aarhus statement: improving design and reporting of studies on early cancer diagnosis. Br J Cancer. 2012;106(7):1262–7. Available from: http://www.ncbi.nlm.nih.gov/pubmed/22415239 10.1038/bjc.2012.68 PMC3314787 22415239

[10] Unger-Saldaña K, Fitch-Picos K, Villarreal-Garza C. Breast Cancer Diagnostic Delays Among Young Mexican Women Are Associated With a Lack of Suspicion by Health Care Providers at First Presentation. J Glob Oncol. 2019;5(5):1–12. Available from: http://www.ncbi.nlm.nih.gov/pubmed/31335236 10.1200/JGO.19.00093 PMC6690634 31335236

[11] Vedsted P, Weller D, Zalounina Falborg A, Jensen H, Kalsi J, Brewster D et al. Diagnostic pathways for breast cancer in 10 International Cancer Benchmarking Partnership (ICBP) jurisdictions: an international comparative cohort study based on questionnaire and registry data. BMJ Open. 2022;12(12):e059669. Available from: https://bmjopen.bmj.com/lookup/doi/10.1136/bmjopen-2021-059669 10.1136/bmjopen-2021-059669 PMC9756230 36521881

[12] Weller D, Menon U, Zalounina Falborg A, Jensen H, Barisic A, Knudsen AK, et al. Diagnostic routes and time intervals for patients with colorectal cancer in 10 international jurisdictions; findings from a cross-sectional study from the International Cancer Benchmarking Partnership (ICBP). BMJ Open. 2018;8(11):e023870. 30482749 10.1136/bmjopen-2018-023870 PMC6278806

[13] Walter F, Webster A, Scott S, Emery J. The Andersen Model of Total Patient Delay: a systematic review of its application in cancer diagnosis. J Heal Serv Res Policy. 2012;17(2):110–8. 10.1258/jhsrp.2011.010113 PMC3336942 22008712

[14] Weller D, Vedsted P, Anandan C, Zalounina A, Fourkala EO, Desai R, et al. An investigation of routes to cancer diagnosis in 10 international jurisdictions, as part of the International Cancer Benchmarking Partnership: survey development and implementation. BMJ Open. 2016;6(7):e009641. 27456325 10.1136/bmjopen-2015-009641 PMC4964239

[15] Menon U, Vedsted P, Zalounina Falborg A, Jensen H, Harrison S, Reguilon I et al. Time intervals and routes to diagnosis for lung cancer in 10 jurisdictions: cross-sectional study findings from the International Cancer Benchmarking Partnership (ICBP). BMJ Open. 2019;9(11):e025895. Available from: https://bmjopen.bmj.com/lookup/doi/10.1136/bmjopen-2018-025895 10.1136/bmjopen-2018-025895 PMC6886977 31776134

[16] RosePW, Rubin G, Perera-Salazar R, Almberg SS, Barisic A, Dawes M, et al. Explaining variation in cancer survival between 11 jurisdictions in the International cancer benchmarking partnership: a primary care vignette survey. BMJ Open. 2015;5(5). 10.1136/bmjopen-2014-007212. 10.1136/bmjopen-2014-007212 PMC4452748 26017370

[17] Jensen H, Tørring ML, Olesen F, Overgaard J, Vedsted P. Cancer suspicion in general practice, urgent referral and time to diagnosis: A population-based GP survey and registry study. BMC Cancer. 2014;14:636. 25175155 10.1186/1471-2407-14-636 PMC4164756

[18] McPhail S, Swann R, Johnson SA, Barclay ME, Abd Elkader H, Alvi R et al. Risk factors and prognostic implications of diagnosis of cancer within 30 days after an emergency hospital admission (emergency presentation): an International Cancer Benchmarking Partnership (ICBP) population-based study. Lancet Oncol. 2022;23(5):587–600. Available from: https://linkinghub.elsevier.com/retrieve/pii/S1470204522001279 10.1016/S1470-2045(22)00127-9 PMC9046095 35397210

[19] Ruco A, Groome PA, McBride ML, Decker KM, Grunfeld E, Jiang L et al. Factors Associated with the Breast Cancer Diagnostic Interval across Five Canadian Provinces: A CanIMPACT Retrospective Cohort Study. Cancers (Basel). 2023;15(2):404. Available from: https://www.mdpi.com/2072-6694/15/2/404 10.3390/cancers15020404 PMC9857089 36672357

[20] Allgar VL, Neal RD. Delays in the diagnosis of six cancers: analysis of data from the National Survey of NHS Patients: Cancer. Br J Cancer. 2005;92(11):1959–70. Available from: https://www.nature.com/articles/6602587 10.1038/sj.bjc.6602587 PMC2361797 15870714

[21] Mavor ME, Groome PA, Asai Y, Langley H, Look Hong NJ, Wright FC et al. Characterising melanoma diagnostic pathways for patients in routine practice using administrative health data in Ontario, Canada: a population-based study. BMJ Open [Internet]. 2025;15(1):e086140. Available from: http://www.pubmedcentral.nih.gov/articlerender.fcgi?artid=PMC11784364" 10.1136/bmjopen-2024-086140 PMC11784364 39890150

[22] de Almeida PF, Giovanella L, Schenkman S, Franco CM, Duarte PO, Houghton N et al. Perspectives for Primary Health Care public policy in South America. Cien Saude Colet. 2024;29(7):e03792024. Available from: http://www.scielo.br/scielo.php?script=sci_arttext&pid=S1413-81232024000700203&tlng=en 10.1590/1413-81232024297.03792024 38958327

[23] Strasser-Weippl K, Chavarri-Guerra Y, Villarreal-Garza C, Bychkovsky BL, Debiasi M, Liedke PER, et al. Progress and remaining challenges for cancer control in Latin America and the Caribbean. Lancet Oncol. 2015;16(14):1405–38. 26522157 10.1016/S1470-2045(15)00218-1

[24] Medeiros GC, Thuler LCS, Bergmann A. Delay in breast cancer diagnosis: a Brazilian cohort study. Public Health. 2019;167:88–95. Available from: 10.1016/j.puhe.2018.10.012 10.1016/j.puhe.2018.10.012 30641460

[25] Barros ÂF, de Araújo JM, Murta-Nascimento C, Dias A. Clinical pathways of breast cancer patients treated in the Federal District, Brazil. Rev Saude Publica. 2019;53(1):1–11. 10.11606/S1518-8787.2019053000406 PMC6390660 30726495

[26] Traldi MC, Galvão P, de Morais SS. Fonseca MRC da C. Demora no diagnóstico de câncer de mama de mulheres atendidas no Sistema Público de Saúde. Cad Saúde Coletiva. 2016;24(2):185–91.

[27] Martínez-Pérez DC, Gómez-Wolff LR, Ossa-Gómez CA, Hernández-Herrera GN, Rivas-Bedoya Y, García-García HI. Asociación entre retraso en el diagnóstico y estadio clínico avanzado de cáncer de mama al momento de la consulta en cuatro centros oncológicos de Medellín, Colombia, 2017. Estudio de corte transversal. Rev Colomb Obstet Ginecol. 2020;71(2):87–102. Available from: https://revista.fecolsog.org/index.php/rcog/article/view/3410 10.18597/rcog.3410 32770869

[28] Foletto EF, Jackisch SE, Dotto ML, Severo C, Pappen E, de Moura Valim AR et al. Therapeutic itinerary of colorectal cancer patients treated in the state of Rio Grande do Sul. J Coloproctology [Internet]. 2016;36(02):091–6. Available from: http://www.thieme-connect.de/DOI/DOI?10.1016/j.jcol.2016.03.008

[29] Vázquez ML, Vargas I, Rubio-Valera M, Aznar-Lou I, Eguiguren P, Mogollón-Pérez AS et al. Improving equity in access to early diagnosis of cancer in different healthcare systems of Latin America: protocol for the EquityCancer-LA implementation-effectiveness hybrid study. BMJ Open. 2022;12(12):e067439. Available from: https://bmjopen.bmj.com/lookup/doi/10.1136/bmjopen-2022-067439 10.1136/bmjopen-2022-067439 PMC9748968 36523219

[30] Giovanella L, de Almeida PF, Romero RV, Oliveira S, Silva HT. [Overview of Primary Health Care in South America: conceptions, components and challenges]. Saude debate. 2015;39(105):301–22.

[31] Ministeriode Salud. Gobierno de Chile. Plan nacional de Cáncer 2022–2027. 2022. https://www.iccp-portal.org/sites/default/files/plans/Plan-de-Accio%CC%81n-Adulto-del-Plan-Nacional-de-Ca%CC%81ncer-2022-2027.pdf.

[32] Ministerio de Salud Pública. Ecuador. Estrategia nacional para la atención integral del cáncer en Ecuador. 2017. Available from: https://aplicaciones.msp.gob.ec/salud/archivosdigitales/documentosDirecciones/dnn/archivos/ac_0059_2017.pdf

[33] Ministerio de Salud y Protección Social. Colombia. Resolución 3280 de 2018 [Internet]. 2018. Available from: https://www.minsalud.gov.co/Normatividad_Nuevo/Resolución No. 3280 de 20183280.pdf.

[34] Repúblicade Colombia. Ley 2194. 2022. https://www.funcionpublica.gov.co/eva/gestornormativo/norma.php?i=177606.

[35] Ministeriode salud y protección social. Colombia. Resolución n^o^10, julio 2024. 2024. https://www.minsalud.gov.co/Normatividad_Nuevo/Circular%20Externa%20No.%20010%20de%202024.pdf.

[36] de Salud M. Chile. Guías Clínicas Auge. 2024. Available from: https://diprece.minsal.cl/le-informamos/auge/acceso-guias-clinicas/guias-clinicas-auge/

[37] Vargas I, Mogollón-Pérez AS, Eguiguren P, Torres AL, Peralta A, Rubio-Valera M, et al. Understanding the health system drivers of delayed cancer diagnosis in public healthcare networks of Chile, Colombia and Ecuador: A qualitative study with health professionals, managers and policymakers. Soc Sci Med. 2025;365:117499. (October 2024). 39626381 10.1016/j.socscimed.2024.117499

[38] Aday LA, Andersen R. A framework for the study of access to medical care. Health Serv Res. 1974;9(3):208–20. 4436074 PMC1071804

[39] LwangaS, Lemeshow S. Sample size determination in health studies: a practical manual. Geneva; 1991. https://iris.who.int/server/api/core/bitstreams/755654cc-c4d6-4bb3-85fb-9a8cc4db50a0/content.

[40] Coxon D, Campbell C, Walter FM, Scott SE, Neal RD, Vedsted P et al. The Aarhus statement on cancer diagnostic research: turning recommendations into new survey instruments. BMC Heal Serv Res. 2018;18(1):677. Available from: http://www.ncbi.nlm.nih.gov/pubmed/30176861 10.1186/s12913-018-3476-0 PMC6174328 30176861

[41] Unger-saldañaK, Peláez-ballestas I, Infante-castañeda C. Development and validation of a questionnaire to assess delay in treatment for breast cancer. 2012;10–2. 10.1186/1471-2407-12-626. 10.1186/1471-2407-12-626 PMC3543238 23272645

[42] Garcia-Subirats I, Vargas I, Mogollón-Pérez AS, De Paepe P, da Silva MRF, Unger JP, et al. Barriers in access to healthcare in countries with different health systems. A cross-sectional study in municipalities of central Colombia and north-eastern Brazil. Soc Sci Med. 2014;106:204–13. 24576647 10.1016/j.socscimed.2014.01.054

[43] Unger-Saldaña K, Infante-Castañeda CB. Breast cancer delay: a grounded model of help-seeking behaviour. Soc Sci Med. 2011 Apr [cited 2020 Mar 12];72(7):1096–104. Available from: http://www.ncbi.nlm.nih.gov/pubmed/21388729 10.1016/j.socscimed.2011.01.022 21388729

[44] World Health Organization. WHO Global Health Expenditure Database. 2020. Available from: https://apps.who.int/nha/database/country_profile/Index/en

[45] OECD. Beating Cancer Inequalities in the EU. OECD Health Policy Studies. 2024. 261 p. Available from: https://www.oecd.org/en/publications/beating-cancer-inequalities-in-the-eu_14fdc89a-en.html

[46] Neal RD, Tharmanathan P, France B, Din NU, Cotton S, Fallon-Ferguson J et al. Is increased time to diagnosis and treatment in symptomatic cancer associated with poorer outcomes? Systematic review. Br J Cancer. 2015;112 Suppl(Suppl 1):S92-107. Available from: http://www.ncbi.nlm.nih.gov/pubmed/25734382 10.1038/bjc.2015.48 PMC4385982 25734382

[47] Din NU, Ukoumunne OC, Rubin G, Hamilton W, Carter B, Stapley S et al. Age and Gender Variations in Cancer Diagnostic Intervals in 15 Cancers: Analysis of Data from the UK Clinical Practice Research Datalink. Katoh M, editor. PLoS One. 2015;10(5):e0127717. 10.1371/journal.pone.0127717 PMC4433335 25978414

[48] Petrova D, Špacírová Z, Fernández-Martínez NF, Ching-López A, Garrido D, Rodríguez-Barranco M et al. The patient, diagnostic, and treatment intervals in adult patients with cancer from high- and lower-income countries: A systematic review and meta-analysis. Suthar AB, editor. PLOS Med. 2022;19(10):e1004110. 10.1371/journal.pmed.1004110 PMC9584443 36264841

[49] Tørring ML, Frydenberg M, Hansen RP, Olesen F, Vedsted P. Evidence of increasing mortality with longer diagnostic intervals for five common cancers: A cohort study in primary care. Eur J Cancer. 2013;49(9):2187–98. Available from: https://linkinghub.elsevier.com/retrieve/pii/S0959804913000889 10.1016/j.ejca.2013.01.025 23453935

[50] Cavicchioli AC, Menossi MJ, Aparecida R, De Lima G. Cancer in children: The diagnostic itinerary. Rev Lat Am Enfermagem. 2007;15(5):1025–32. Available from: https://www.eerp.usp.br/rlae 10.1590/s0104-11692007000500022 18157458

[51] Tejada-Tayabas LM, Salcedo LA, Espino JM. Medical therapeutic itineraries of women with breast cancer diagnosis affiliated to the people’s health insurance in San Luis Potosí, central Mexico. Cad Saude Publica. 2015;31(1):60–70. 10.1590/0102-311x00009114. 25715292 10.1590/0102-311x00009114

[52] Campaña C, Cabieses B, Obach A, Vezzani F. Healthcare should be the same for everyone: perceived inequities in therapeutic trajectories of adult patients with lung cancer in Chile, a qualitative study. Front Public Heal. 2023;11. 10.3389/fpubh.2023.1228304 PMC10468573 37663832

[53] Uribe Pérez CJ, Amado Niño AM, Rueda Patiño AM, Mantilla Villabona LY. Barreras para la atención en salud del cáncer gástrico, Santander, Colombia. Etapa exploratoria. Rev Colomb Gastroenterol. 2019;34(1):17.

[54] FONASA.FONASA datos abiertos. Estadísticas interactivas. Población beneficiaria FONASA a Diciembre 2024. 2025. https://share.google/J3VV0HUM8VjaK0w0y.

[55] Pham TM, Gomez-Cano M, Salika T, Jardel D, Abel GA, Lyratzopoulos G. Diagnostic route is associated with care satisfaction independently of tumour stage: Evidence from linked English Cancer Patient Experience Survey and cancer registration data. Cancer Epidemiol. 2019;61(March):70–8. Available from: 10.1016/j.canep.2019.04.011 Additional material 1) File name: Additional File 1 File format: Word 365 EquityCancer-LA Questionnaire (English version) 2) File name: Additional File 2 File format: Word 365. 10.1016/j.canep.2019.04.011 31153049

