# Supplementary material for: Delays and detours during cancer diagnosis: a cross-sectional study on patient pathways and provider intervals in public healthcare networks of Chile, Colombia and Ecuador
Source: BMC Cancer. 2026 Feb 26;26:438. doi: 10.1186/s12885-026-15737-5 (PMC13059593; doi:10.1186/s12885-026-15737-5)
Supplement: Supplementary file 1 — Additional file 1. EquityCancer-LA Questionnaire (English version) [file 12885_2026_15737_MOESM1_ESM.pdf]

QUESTIONNAIRE ON ACCESS TO CANCER DIAGNOSIS*
(EQUITYCANCER-LA)
COUNTRY (*Introduce*)

GENERAL INFORMATION

The (*complete*), in collaboration with (*complete*), is conducting a study with the aim of analyzing the delay in cancer diagnosis and the main barriers in the use of health services during diagnostic process, as well as, identifying areas for improvement.

Thus, a survey is being carried out to learn about patients' experiences of health services during cancer diagnosis.

The information you provide is very important, as it will help identify existing problems in health services and suggest changes to improve early cancer diagnosis. The results of the survey will be subsequently disclosed. They will be processed in an aggregated form to ensure the confidentiality of the data. This implies that your responses will be anonymous. Your participation in this study will have no effect on your treatment, health benefits, legal matters, or similar issues.

Individuals using the information you provide are legally obligated to maintain the confidentiality of both the data and the respondent. That is, they will not use it for any purpose other than those described above, so we can guarantee the confidentiality of your information.

Resolution/law on data protection (to be completed) of (to be completed), Ministry of Health

We appreciate your collaboration

DATA FROM THE APPLICATION OF THE QUESTIONNAIRE

Interviewer:____

Date of the survey ___ ___ / ___ ___ / ___ ___ ___ ___
D D / M M / Y Y Y Y

Start time: ___ ___: ___ ___

Health centre where the respondent was selected: ______________________________ __ __ __

Locality (*Adapt*): ______________________________________ __ __ __

START OF THE INTERVIEW

Before we start the interview, how would you like me to address you? (___________________).

Now I am going to ask you some questions about the use of health services during the diagnosis of your illness.

→ Can you confirm that you currently have or have had cancer? ___________ → *Fill in according to the diagnosis on the patient's record*

Copyright 2022©. María-Luisa Vázquez, Ingrid Vargas (Consortium for Health Care and Social Services of Catalonia, Spain); Pamela Eguiguren (Escuela de Salud Pública Dr. Salvador Allende Gossens, Facultad de Medicina, Universidad de Chile, Chile); Amparo-Susana Mogollón-Pérez (Universidad del Rosario, Colombia); Ana-Lucía Torres and Andrés Peralta (Instituto de Salud Pública, Pontificia Universidad Católica del Ecuador, Ecuador); Maria Rubio-Valera and Ignacio Aznar-Lou (Sant Joan de Déu Research Foundation, Spain); Signe Smith Jervelund (University of Copenhagen, Denmark); Sónia Dias (NOVA National School of Public Health, NOVA University Lisbon, Portugal). "Improving equity in access to early diagnosis of cancer: implementation research in different healthcare systems of Latin America (EquityCancer-LA)". This project has received funding from the European Union's Horizon 2020 research and innovation programme under grant agreement No 965226 on the call topic SC1-BHC-17-2020, Global Alliance for Chronic Diseases - Prevention and/or early diagnosis of cancer. The full or partial reproduction of this work is authorized, provided that the authors and copyright holders are explicitly credited.

*Authors translation from Spanish

1. PERCEPTION OF THE HEALTH PROBLEM AND CARE-SEEKING	
**1.1 ¿ How did you become aware you had this health problem?** → ***Read** the response options*<br>**(1)** Symptoms or discomfort<br>**(2)** Routine medical examination/screening - Public healthcare service→***q.1.5***<br>**(3)** Routine medical examination/screening - Private healthcare service→***q.1.5***<br>**(4)** I was examined for another problem (casual finding) - Public healthcare service→***q.1.5***<br>**(5)** I was examined for another problem (casual finding) - Private healthcare service→***q.1.5***<br>**(6)** Other, which one? ……………………………………………→***q.1.5***<br>*[SPECIFY]*	___ ___
**1.2 What symptoms or discomfort did you noticed?**<br>____<br>____<br>____<br>____	___ ___<br>___ ___<br>___ ___<br>___ ___
**1.2.1 Can you tell me when you noticed the symptoms or discomfort?** → ***Write the answer** and continue with the questions if the date is not complete.*<br>___ ___ / ___ ___ / ___ ___ ___ ___<br>D D / M M / Y Y Y Y<br>→ *Can you tell me the month and year?*<br>→ *If he/she does not remember them, can you tell me how long ago it was?*<br>____<br>→ *If he/she does not remember anything mark* **(99)**	___ ___ ___ ___<br>[*DAYS*]
**1.2.2 <u>Which health service did you first go to attend your symptoms or discomfort</u>?** → ***Read** response options (Adapt to each country)*<br>**(1)** Health center — **(2)** Emergency service<br>**(3)** Polyclinic — **(4)** Public Hospital<br>**(5)** Private clinic — **(6)** Private emergency service<br>**(7)** Private hospital — **(8)** Other, which one?................................<br>*[SPECIFY]*	___ ___
**1.3 <u>Can you tell me when you made the appointment (or went to the emergency service)</u>?** → ***Write the answer** and continue with the questions if the date is not complete.*<br>___ ___ / ___ ___ / ___ ___ ___ ___<br>D D / M M / Y Y Y Y<br>→ *Can you tell me the month and year?*<br>→ *If you he/she does not remember them, can you tell me how long ago it was?*<br>____<br>→ *If he/she does not remember anything mark* **(99)**	___ ___ ___ ___<br>[*DAYS*]
**1.3.1 <u>Did you receive attention on that occasion?</u>**<br>**(1)** Yes→***q.1.3.2*** **(2)** No **(98)** DK/NA →***q.1.4***	___ ___
**1.3.1.1 Why were you not attended to?**<br>____<br>____<br>____<br>____<br>→ ***Go to q.1.4***	___ ___<br>___ ___<br>___ ___<br>___ ___
**1.3.2 ¿ Which professional attended you on that occasion?** *(Add list of professionals according to country)*<br>**(1)** General doctor — **(2)** Specialist<br>**(3)**_______________ — **(4)**__________________<br>**(5)**_______________ — **(98)** DK/NA	___ ___
**1.4 What did you think about the time that passed from when you first noticed your symptoms or discomfort <u>until you requested an appointment (or went to the emergency room)</u>?** → ***Read** response options*<br>**(1)** Short→***Section 2*** **(2)** Normal→***Section 2*** **(3)** Long **(98)** DK/NA→***Section* 2**	___ ___

**1.4.1 Why did you delay in requesting an appointment with the health service (or going to the emergency service)?**<br>______________________________<br>______________________________<br>______________________________<br>→ ***Go to section 2***	___ ___<br>___ ___<br>___ ___
**1.5 Can you tell me when you had the screening/medical exploration or …………………….?** → ***Fill in*** *according to answer in q.1.1*<br>→ ***Write the answer*** *and continue with the questions if the date is not complete.*<br>___ ___ / ___ ___ / ___ ___ ___ ___<br>D D / M M / Y Y Y Y<br>→ *Can you tell me the month and year?*<br>→ *If he/she does not remember them, can you tell me how long ago it was?*<br>______________________________<br>→ *If he/she does not remember anything mark* **(99)**	___ ___ ___ ___<br>*[DAYS]*
**1.6 After the screening or medical examination, were you referred to a doctor in a public health service?**<br>**(1)** Yes **(2)** No→ ***q.1.6.2*** **(98)** DK/NA →***q.1.6.2***	___ ___
**1.6.1 To which doctor were you referred to?** → ***Read*** *the response options* (Adapt to each country)<br>**(1)** General doctor at the health center<br>**(2)** Specialist doctor at the hospital<br>**(3)** Other, which one? ……………………………………………………….<br>*[SPECIFY]*	___ ___
**1.6.2 Can you tell me when you requested the medical appointment (with public health services) after the screening/medical examination?** → ***Write the answer*** *and continue with the questions if the date is not complete.*<br>___ ___ / ___ ___ / ___ ___ ___ ___<br>D D / M M / Y Y Y Y<br>→ *Can you tell me the month and year?*<br>→ *If he/she does not remember them, can you tell me how long it has been since you requested the appointment?*<br>______________________________<br>→ *If he/she does not remember anything, mark* **(99)**	___ ___ ___ ___<br>*[DAYS]*

→ ***Read.*** *Next, I will ask you some questions to learn about the health services you used until you were diagnosed with cancer.*

2. USE OF HEALTH SERVICES UNTIL CANCER DIAGNOSIS

2.1 What services did you use from the time you noticed symptoms or discomfort (or were screened) until you were diagnosed with cancer? (Adapt for each country**).**

(1) General doctor at the health center
(2) Emergency service
(3) Emergency service of National Cancer Institute
(4) Specialist
(5) Specialist of National Cancer Institute
(6) Hospitalization
(7) National Cancer Institute hospitalization
(8) Private general doctor
(9) Private emergency service
(10) Private specialist
(11) Private hospitalization
(12) Private laboratory or imaging center
(13) Other, which one?.................................
[SPECIFY]

→***Write down*** *in chronological order the services he/she attended as many times as they are mentioned.*

**2.2 Where was your cancer diagnosis confirmed (public service)?** →***Write*** *the name or address of the center*<br>______________________________	___ ___

**2.3 How long was it from your first doctor's appointment or emergency visit until your cancer diagnosis was confirmed?**<br>____	___ ___ ___<br>*[DAYS]*
**2.3.1 What did you think of this time?** → ***Read*** *the response options*<br>**(1)** Short→ ***q.2.3.2*** **(2)** Normal→ ***q.2.3.2*** **(3)** Long **(98)** DK/NA→ ***q.2.3.2***	___ ___
**2.3.1.1 Do you think this time affected your health?**<br>**(1)** Yes **(2)** No→ ***q.2.3.2*** **(98)** DK/NA→ ***q.2.3.2***	
**2.3.1.1.1 How do you think this affected your health?**<br>____<br>____<br>____	___ ___<br>___ ___<br>___ ___
**2.3.2 During the investigation of the symptoms, <u>did you have to stop working, studying or doing household</u> chores because of this health problem?**<br>**(1)** Yes **(2)** No→ ***q.2.4*** **(98)** DK/NA → ***q.2.4***	___ ___
**2.3.3 How many days did you have to stop working, studying or doing household chores?**<br>____	___ ___ ___<br>*[DAYS]*
**2.4 Did you or your family need financial help or a loan for your cancer diagnosis?**<br>*TO BE ADAPTED TO EACH COUNTRY*<br>**(1)** Yes **(2)** No→ ***Next section*** **(98)** DK/NA→ ***Next section***	___ ___
**2.5 What type of financial help/loan did you request (receive)?** → ***Read*** *the response options TO BE ADAPTED TO EACH COUNTRY*<br>**(1)** Family members or others donated money to help cover the diagnosis<br>**(2)** Sale of personal or family property<br>**(3)** Charity fundraiser (raffle, bingo, etc.)<br>**(4)** Use of savings<br>**(5)** Loan from family and/or friends<br>**(6)** Loan from a bank or other financial institution<br>**(7)** Other, which one? __________________________<br>*[SPECIFY]*	___ ___
→ ***Complete*** *the sections corresponding to 3 to 6, following the order of question 2.1.*	

→ ***Read.*** *Next, I will ask you some questions about your experience with health services for the cancer diagnosis, beginning with the first place you visited.*

3. EXPERIENCE IN THE HEALTH SERVICES FOR DIAGNOSIS: GENERAL DOCTOR

→ ***ASK*** *only if you consulted the general doctor at the public health center of the network for cancer diagnosis*

**3.1 At your first consultation with the general practitioner for investigating your symptoms, which health center did you visit?** → ***Write*** *the name or address of the center*<br>____	___ ___
**3.1.1 How was your first consultation with the general doctor?** → ***Read*** *the response options.*<br>**(1)** In-person **(2)** By phone **(3)** Video consultation **(98)** DK/NA	___ ___
**3.1.2 Can you tell me when this first consultation with the general doctor for investigating your symptoms took place?**→ ***Write the answer*** *and continue with the questions if the date is not complete.*<br>___ ___ / ___ ___ / ___ ___ ___ ___<br>D D / M M / Y Y Y Y<br>→ *Can you tell me the month and year?*<br>→ *If he/she does not remember them, can you tell me how long it has been since <u>your first consultation</u>?*<br>____<br>→ *If he/she does not remember anything mark* **(99)**	___ ___ ___ ___<br>*[DAYS]*
**3.2 How long did it take from <u>the time you requested the appointment to the time you were seen</u>?**<br>____	___ ___ ___<br>*[DAYS]*

**3.3 <u>Did you have to pay for the consultation?</u>**<br>**(1)** Yes **(2)** No→***q.3.4*** **(98)** DK/NA →***q.3.4***	___ ___
**3.3.1 How much were you charged?**<br>____$<br>→ *If he/she does not remember how much, mark* **(99)**	___ ___ ___
**3.4 What means of transport did you use to get to the health center?** →*Several response options*<br>**(1)** On foot/by bicycle→***q.3.5*** **(2)** Bus **(3)** Own vehicle **(4)** Taxi<br>**(5)** Other, which one? ____________________<br>**(96)** N/A-Telephone/video-consultation→***q.3.6***	___ ___
**3.4.1 <u>How much did you have to pay for round-trip transportation</u>?**→ ***Ask*** *the person to take into account all transportation costs including those of any accompanying person*<br>____$<br>→ *If he/she does not remember how much you had to pay mark* **(99)**	___ ___ ___
**3.5 <u>How long did it take you to get to the health center?</u>**<br>____	___ ___<br>[*MINUTES*]
**3.6 <u>How many times did you consult the general doctor for your symptoms</u>?**<br>____<br>→ *If the answer is only once, skip to q.3.8.*	___ ___
**3.7 During consultations with the general doctor, were you seen by the same doctor?**→ ***Read*** *response options.*<br>**(1)** Always **(2)** Often **(3)** Rarely **(4)** Never **(98)** DK/NA	___ ___
**3.8 Did the general doctors prescribe any medication to treat your symptoms or discomfort?**<br>**(1)** Yes **(2)** No→***p.3.9*** **(98)** DK/NA →***p.3.9***	___ ___
**3.8.1 Did you have to complete any procedures to get these medications authorized?** (***ONLY COLOMBIA***)<br>**(1)** Yes **(2)** No **(98)** DK/NA	___ ___
**3.8.2 <u>Did you pay for these drugs</u>?**<br>**(1)** Yes **(2)** No→***p.3.8.4*** **(3)** Partially **(98)** DK/NA →***p.3.8.4***	___ ___
**3.8.3 <u>How much were you charged</u> for these drugs?**→ ***Ask the person*** *to consider all drugs.*<br>____$<br>→ *If he/she does not remember how much, mark* **(99)**	___ ___ ___
**3.8.3.1 Why did you have to pay?**<br>____<br>____	___ ___<br>___ ___<br>___ ___
**3.8.4 Did you have difficulties to get these drugs?**<br>**(1)** Yes **(2)** No→***p.3.9*** **(98)** DK/NA→***p.3.9***	___ ___
**3.8.4.1 What difficulties did you have?**<br>____<br>____<br>____	___ ___<br>___ ___<br>___ ___
**3.9 Did the general doctors <u>order tests</u> for the investigation of your symptoms?**<br>**(1)** Yes **(2)** No→***p.3.11*** **(98)** DK/NA →***p.3.11***	___ ___
**3.9.1 What tests were ordered?** → *If he/she does not remember them, read the list of tests according to cancer site. Several response options.*<br>*(List of most common tests that can be ordered at primary care for the selected cancers in each country. Sort list by general tests and cancer site)*<br>**(1)**______________ **(2)**______________<br>**(3)**______________ **(4)**______________<br>**(5)**______________ **(6)** Other, which one? ____________________<br>**(98)** DK/NA	___ ___

**3.9.2 Did you have to complete any procedures to get any of the tests authorized?**<br>**(1)** Yes **(2)** No **(98)** DK/NA	___ ___
**3.9.3 What means of transportation did you use to get to the tests?** ***→Several response options***<br>**(1)** On foot/bicycle→***p.3.10*** **(2)** Bus **(3)** Own vehicle **(4)** Taxi<br>**(5)** Other, which one? ___________________________	___ ___
**3.9.3.1 <u>How much did you have to pay for round-trip transportation?</u> →** ***Ask the person** to account for all transport costs for ALL tests that had to be performed, including those of the accompanying person.*<br>____$<br>→ *If he/she does not remember how much, mark* **(99)**	___ ___
**3.10 Did you have any difficulty undergoing any of these tests ordered by the general doctors?**<br>**(1)** Yes **(2)** No→***p.3.11*** **(98)** DK/NA→***p.3.11***	___ ___
**3.10.1 What difficulties?**<br>____<br>____<br>____	___ ___<br>___ ___<br>___ ___
**3.10.1.1 Do you think these difficulties affected your health?**<br>**(1)** Yes **(2)** No→***p.3.11*** **(98)** DK/NA→***p.3.11***	___ ___
**3.10.1.2 How do you think they affected your health?**<br>____<br>____<br>____	___ ___<br>___ ___<br>___ ___
**3.11 During your last visit to the general doctor, <u>what did he/she say you might have</u>?**<br>**(1)** Benign tumor **(2)** Tumor (suspicious)<br>**(3)** Need to be further explored **(4)** Nothing<br>**(5)** Malignant tumor (cancer) **(6)** Other, which one?_____________	___ ___
**3.11.1 Can you tell me <u>when you were informed of this diagnosis</u>?** → ***Write the answer** and continue with the questions if the date is not complete.*<br>___ ___ / ___ ___ / ___ ___ ___ ___<br>D / M / Y Y Y Y<br>→ *Can you tell me the month and year?*<br>→ *If you he/she does not remember them, can you tell me how long it has been since <u>the general doctor informed you of this diagnosis</u>?*<br>____<br>→ *If he/she does not remember anything mark* **(99)**	___ ___ ___ ___<br>[*DAYS*]
**3.12 During this visit with the general doctor, <u>did he/she refer you to a specialist for further investigation of your symptoms</u>?**<br>**(1)** Yes **(2)** No→***p.3.15*** **(98)** DK/NA →***p.3.15***	___ ___
**3.12.1 Which specialist were you referred to?** →***If he/she does not remember, read** the response options according to cancer site. (Add list of professionals according to the country)*<br>**(1)**_______________ **(2)**__________________<br>**(3)**_______________ **(4)**__________________<br>**(5)**_______________ **(5) Other, which one?** __________________<br>**(98)** DK/NA	___ ___
**3.13 Did the general practitioner give you a <u>report for the specialist</u>?** (*Adapt/apply according to country*)<br>**(1)** Yes **(2)** No **(98)** DK/NA	___ ___
**3.14 After the referral, <u>what type of information did you receive</u>?** → *If he/she does not remember, read the response options*<br>**(1)** Date and time of the appointment **(2)** Name of the center/hospital<br>**(3)** Address of the center **(4)** Directions to the center/hospital<br>**(5)** Payments to be made **(6)** Name of the doctor<br>**(7)** Other, which one? ________________ **(97)** None<br>**(98)** DK/NA	___ ___
**3.15 During the symptom investigation, <u>were you ever unable to attend (or complete) a scheduled consultation with the general doctor</u>?**<br>**(1)** Yes **(2)** No→***p.3.16*** **(98)** DK/NA→***p.3.16***	___ ___

**3.15.1 Why were you unable to attend/complete the consultation?**<br>____________________<br>____________________<br>____________________	__ __<br>__ __<br>__ __
**3.15.2 Do you think that not being able to attend (complete) the consultation affected your health?**<br>**(1)** Yes **(2)** No→***q.3.16*** **(98)** DK/NA →***q.3.16***	__ __
**3.15.3 How do you think it affected your health?**<br>____________________<br>____________________<br>____________________	__ __<br>__ __<br>__ __
**3.16 During the symptom investigation, did you ever have a scheduled consultation, <u>and the general doctor did not attend you</u>?**<br>**(1)** Yes **(2)** No→***q.3.17*** **(96)** NA→***q.3.17*** **(98)** DK/NA→***q.3.17***	__ __
**3.16.1 Why didn't the general doctor attend to you?**<br>____________________<br>____________________<br>____________________	__ __<br>__ __<br>__ __
**3.16.2 Do you think not being attended to <u>affect your health</u>?**<br>**(1)** Yes **(2)** No→***q.3.17*** **(98)** DK/NA →***q.3.17***	__ __
**3.16.3 How do you think it affected your health?**<br>____________________<br>____________________<br>____________________	__ __<br>__ __<br>__ __
**3.17 Before or during the investigation of your symptoms with the general doctor, <u>did you consult a private doctor</u>?**<br>**(1)** Yes **(2)** No→***q.3.18*** **(98)** DK/NA→***q.3.18***	__ __
**3.17.1 Why did you use a private health service?**<br>____________________<br>____________________<br>____________________	__ __<br>__ __<br>__ __
**3.18 Before or during the investigation of your symptoms with the general doctor, <u>did you have private testing</u>?**<br>**(1)** Yes **(2)** No→ ***Next section*** **(98)** DK/NA→***Next section***	__ __
**3.18.1 <u>How much were you charged for the tests</u>?** →***Consider** all tests*<br>____________________$<br>→ *If he/she does not remember how much, mark* **(99)**	__ __
**3.18.2 Why did you get tested in a private health service?**<br>____________________<br>____________________<br>____________________	__ __<br>__ __<br>__ __

4. EXPERIENCE IN HEALTH SERVICES FOR DIAGNOSIS: SPECIALIST

→ ***ASK*** *only if he/she consulted a specialist of a public health service of the network for cancer diagnosis as indicated in section 2, question 3.12 or 5.12.2.*

**4.1. In your first consultation with a specialist for the investigation of your symptoms, which hospital did you visit?** →***Write*** *the name of the center or its address*<br>______________________________	___ ___
**4.1.1 How was your first consultation with the specialist?** → ***Read*** *the answer options*<br>**(1)** In-person **(2)** Telephone **(3)** Video consultation **(98)** DK/NA	___ ___
**4.1.2 Can you tell me when this first consultation with the specialist for investigating your symptoms took place?** → ***Write the answer*** *and continue with the questions if the date is not complete.*<br>____ ___ / ___ ___ / ___ ___ ___ ___<br>D D / M M / Y Y Y Y<br>→*¿Can you tell me the month and year?*<br>→ *If he/she does not remember,* ¿can you tell how long it has been since your first consultation?<br>____________<br>→*If he/she does not remember, mark* **(99)**	___ ___ ___ ___<br>[*DAYS*]
**4.1.3 What type of specialist attended you in that first consultation?** *(Adapt according to the most frequent cancers selected in each country).* → ***Read*** *the answer options according to cancer stie.*<br>**(1)**______________ **(2)**______________<br>**(3)**______________ **(4)**______________<br>**(5)**______________ **(5)** Other, which one? ______________<br>**(98)** DK/NA	___ ___
**4.2 How did you get the appointment?**→ ***Read*** *the answer options*<br>**(1)** Referral from general doctor **(2)** Referral from emergency service<br>**(3)** Referral from another specialist **(4)** Own initiative<br>**(5)** Screening **(6)** Other, which one?______________	___ ___
**4.3 How long did it take from the time you requested the appointment to the consultation?**<br>______________________________	___ ___ ___<br>[*DAYS*]
**4.4 Did you have to complete any procedures to get authorization for the specialist consultation?** (N/A in Ecuador)<br>**(1)** Yes **(2)** No **(98)** DK/NA	___ ___
**4.5 Did you have to pay for the consultation?**<br>**(1)** Yes **(2)** No →***q.4.6*** **(98)** DK/NA →***q.4.6***	___ ___
**4.5.1 How much were you charged?**<br>______________________________**$**<br>→ *If he/she does not remember how much, mark* **(99)**	___ ___
**4.6 What means of transport did you use to get to the specialist?** →*Several response options*<br>**(1)** On foot/bicycle →***q.4.7*** **(2)** Bus **(3)** Own vehicle **(4)** Taxi<br>**(5)** Other, which one? ______________**(96)** N/A-Telephone/video-consultation→***q.4.8***	___ ___
**4.6.1 How much did you have to pay for round-trip transportation?** → *Ask the person to take into account all transport costs including those of any accompanying person.*<br>______________________________**$**<br>→ *If he/she does not remember how much, mark* **(99)**	___ ___
**4.7 How long did it take you to get to the specialist?**<br>______________________________	___ ___ ___<br>[*MINUTES*]
**4.8 How many times did you consult the specialist during the investigation of your symptoms?**<br>______________________________	___ ___
**4.9 Did the specialists prescribe any medication during the investigation of your symptoms?**<br>**(1)** Yes **(2)** No →***q.4.10*** **(98)** DK/NA →***q.4.10***	___ ___
**4.9.1 Did you have to complete any procedures to get these medications authorized?** (Chile and Colombia)<br>**(1)** Yes **(2)** No **(98)** DK/NA	___ ___

**4.9.2 Did you pay for these drugs?**<br>**(1)** Yes **(2)** No→***q.4.9.5*** **(3)** Partially **(98)** DK/NA→***q.4.9.5***	__ __
**4.9.3 How much were you charged for these drugs?**<br>______________________________________$<br>→ *If he/she does not remember how much, mark* **(99)**	__ __ __
**4.9.4 Why did you have to pay?**<br>______________________________________<br>______________________________________<br>______________________________________	__ __<br>__ __<br>__ __
**4.9.5 Did you have any difficulties to get these drugs?**<br>**(1)** Yes **(2)** No→***q.4.10*** **(98)** DK/NA →***q.4.10***	__ __
**4.9.5.1 What difficulties did you have?**<br>______________________________________<br>______________________________________<br>______________________________________	__ __<br>__ __<br>__ __
**4.10 Did the specialists <u>order tests</u> for the investigation of your symptoms?**<br>**(1)** Yes **(2)** No → ***q.4.12*** **(98)** DK/NA →***q.4.12***	__ __
**4.10.1 What tests were ordered?** →*If he/she does not remember them, read the list of tests according to type of cancer. Several response options*<br>*(List of most common tests that can be ordered by specialists for the selected cancers in each country. Sort by general tests and cancer site)*<br>**(1)**________________ **(2)**___________________<br>**(3)**________________ **(4)**___________________<br>**(5)**________________ **(6)** Other, which one? ______________________<br>**(98)** DK/NA	__ __
**4.10.2 <u>Did you have to complete any procedures</u> to get any of these tests authorized?**<br>**(1)** Yes **(2)** No **(98)** DK/NA	__ __
**4.10.3 What <u>means of transportation</u> did you use to get to the tests?** →*Several response options*<br>**(1)** On foot/bicycle→***q.4.11*** **(2)** Bus **(3)** Own vehicle **(4)** Taxi<br>**(5)** Other, which one? ___________________________	__ __
**4.10.3.1 How much did you have to pay for round-trip transportation?**→ *Ask the person to take into account all transport costs for ALL tests that had to be performed and include those of the accompanying person.*<br>______________________________________$<br>→ *If he/she does not remember how much, mark* **(99)**	__ __
**4.11 Did you have any <u>difficulty undergoing any of these tests</u> ordered by specialists?**<br>**(1)** Yes **(2)** No→***q.4.12*** **(98)** DK/NA→***q.4.12***	__ __
**4.11.1 What difficulties?**<br>______________________________________<br>______________________________________<br>______________________________________	__ __<br>__ __<br>__ __
**4.11.1.1 Do you think these difficulties affected your health?**<br>**(1)** Yes **(2)** No→***q.4.12*** **(98)** DK/NA→***q.4.12***	__ __
**4.11.1.2 How did they affected your health?**<br>______________________________________<br>______________________________________<br>______________________________________	__ __<br>__ __<br>__ __
**4.12 Finally, what did the specialists tell you that you had?** → ***Read** the answer options*<br>**(1)** Benign tumour→***q.4.15*** **(2)** Suspicious tumour→***q.4.15***<br>**(3)** Malignant tumor (cancer) **(4)** Other, which one?________________→***q.4.15***	__ __

**4.12.1 Can you tell me when you were informed of this diagnosis? (confirmed diagnosis)** → ***Write the answer*** *and continue with the questions if the date is not complete.*<br>___ ___ / ___ ___ / ___ ___ ___ ___<br>D D / M M / Y Y Y Y<br>→*Can you tell me the month and year?*<br>→*If he/she does not remember them, can you tell me how long it has been since you received the diagnosis?*<br>____<br>→*If he/she does not remember anything mark* **(99)**	__ __ __ __<br>[*DAYS*]
**4.13 Did the specialist prescribe you the treatment to follow?**<br>**(1)** Yes→***p.4.15*** **(2)** No **(98)** DK/NA	
**4.14 Did the specialist refer you to another specialist for your cancer treatment?**<br>**(1)** Yes **(2)** No→***q.4.15*** **(98)** DK/NA→***q.4.15***	__ __
**4.14.1 Can you tell me to which specialist you were referred to?** (*Add list of professionals by country*)<br>→ ***Read*** *the response options according to cancer site.*<br>**(1)**______________ **(2)**____________<br>**(3)**______________ **(4)**____________<br>**(5)**______________ **(98)** DK/NA	__ __
**4.15 During the investigation of the symptoms, were you ever unable to attend (or complete) the consultation with the specialist?**<br>**(1)** Yes **(2)** No → ***q.4.16*** **(98)** DK/NA → ***q.4.16***	__ __
**4.15.1 Why were you unable to attend/complete the consultation?**<br>____<br>____<br>____	__ __<br>__ __<br>__ __
**4.15.2 Do you think that not being able to attend (complete) the consultation affected your health?**<br>**(1)** Yes **(2)** No→***q.4.16*** **(98)** DK/NA →***q.4.16***	__ __
**4.15.3 How do you think it affected your health?**<br>____<br>____<br>____	__ __<br>__ __<br>__ __
**4.16 During the symptom investigation, did you ever have a schedule consultation and the specialist did not attend you?**<br>**(1)** Yes **(2)** No →***q.4.17*** **(98)** DK/NA →***q.4.17***	__ __
**4.16.1 Why didn't the specialist attend to you?**<br>____<br>____<br>____	__ __<br>__ __<br>__ __
**4.16.2 Do you think not being attended to affect your health?**<br>**(1)** Yes **(2)** No→***q.4.17*** **(98)** DK/NA→***q.4.17***	__ __
**4.16.3 How do you think it affected your health?**<br>____<br>____<br>____	__ __<br>__ __<br>__ __
**4.17 During the symptom investigation with the specialist, did you consult a private specialist?**<br>**(1)** Yes **(2)** No→***q.4.18*** **(98**) DK/NA→***q.4.18***	__ __
**4.17.1 Why did you use a private health service?**<br>____<br>____<br>____	__ __<br>__ __<br>__ __
**4.18 During the investigation of the symptoms with the specialist, did you have private testing?**<br>**(1)** Yes **(2)** No→***Next section*** **(98)** DK/NA→***Next section***	__ __

**4.18.1 How much were you charged for the tests?** → *Ask the person to take into account all the tests.*<br>______________________________________ **$**<br>→ *If he/she does not remember how much, mark* **(99)**	___ ___
**4.18.2 Why did you get tested in a private health service?**<br>______________________________________<br>______________________________________<br>______________________________________	___ ___<br>___ ___<br>___ ___

**5. EXPERIENCES IN HEALTH SERVICES FOR DIAGNOSIS: EMERGENCY**	
→ ***ASK*** *only if he/she consulted an emergency service of the public health system of the network during the cancer diagnosis as indicated in section 2.*	
**5.1 When you went to the emergency department for your health problem, which center or emergency service did you visit?** → *Write the name of the center or its address. If he/she went several times, ask about the first time.*<br>______________________________________	___ ___
**5.1.1 Can you tell me when you went to the emergency department?** → ***Write the answer*** *and continue with the questions if the date is not complete*<br>___ ___ / ___ ___ / ___ ___ ___ ___<br>D D / M M / Y Y Y Y<br>→*Can you tell me the month and year?*<br>→ *If he/she does not remember them, can you tell me how long it has been since you attended?*<br>______________________________________<br>→ *If he/she does not remember anything mark* **(99)**	___ ___ ___ ___<br>*[DAYS]*
**5.2 Who made the decision to go to the emergency department?** → ***Read*** *the response options*<br>**(1)** Yourself **(2)** Family member or accompanying person<br>**(3)** General doctor at the health centre→***q.5.4*** **(4)** Another, Which one? ________________	___ ___
**5.3 Why didn't you go to general doctor at your health center?**<br>______________________________________<br>______________________________________	___ ___<br>___ ___<br>___ ___
**5.4 Did you have to pay to be seen in the emergency department?**<br>**(1)** Yes **(2)** No→***q.5.5*** **(98)** DK/NA→***q.5.5***	___ ___
**5.4.1 ¿How much were you charged?**<br>______________________________________$<br>→ *If he/she does not remember how much, mark* **(99)**	___ ___ ___
**5.5 What means of transportation did you use to go to the emergency department?** →*Several response options*<br>**(1)** On foot/bicycle→***q.5.6*** **(2)** Bus **(3)** Own vehicle **(4)** Taxi<br>**(5)** Other, which one? ______________________________________	___ ___<br>___ ___
**5.5.1 How much did you have to pay for round-trip transportation?** → ***Ask*** *the person to take into account all transport costs including those of any accompanying person.*<br>______________________________________$<br>→ *If he/she does not remember how much, mark* **(99)**	___ ___
**5.6 How long did it take you to get to the emergency service?**<br>______________________________________	___ ___ ___<br>*[MINUTES]*
**5.7 How many times did you visit the emergency service?**<br>______________________________________	___ ___
**5.8 On any of these occasions, were you hospitalized?**<br>**(1)** Yes→***Go to section 6*** **(2)** No **(98)** DK/NA	___ ___

**5.9 Did the emergency doctors <u>prescribe any medication</u> to treat your symptoms or discomfort?**<br>**(1)** Yes **(2)** No→***q.5.10*** **(98)** DK/NA →***q.5.10***	__ __
**5.9.1 Did you have to complete any procedures to <u>get these medications authorized</u>?** (***ONLY COLOMBIA***)<br>**(1)** Yes **(2)** No **(98)** DK/NA	__ __
**5.9.2 <u>Did you pay for these drugs?</u>**<br>**(1)** Yes **(2)** No→***q.5.9.4*** **(3)** Partially **(98)** DK/NA →***q.5.9.4***	__ __
**5.9.3 How much were you charged for these drugs?**<br>____$<br>→ *If he/she does not remember how much, mark* **(99)**	__ __ __
**5.9.3.1 Why did you have to pay?**<br>____<br>____<br>____	__ __<br>__ __<br>__ __
**5.9.4 <u>Did you have difficulties to get these drugs</u>?**<br>**(1)** Yes **(2)** No→***q.5.10*** **(98)** DK/NA →***q.5.10***	__ __
**5.9.4.1 What difficulties did you have?**<br>____<br>____<br>____	__ __<br>__ __<br>__ __
**5.10 Did the emergency doctor <u>order tests</u> for the investigation of your symptoms?**<br>**(1)** Yes **(2)** No→***q.5.12*** **(98)** DK/NA→***q.5.12***	__ __
**5.10.1 What tests were ordered?** → *If he/she does not remember them, read the list of tests according to cancer site. Several response options.*<br>*(List of most common tests that can be ordered in the emergency department for the selected cancers in each country. Sort by general tests and by cancer site.)*<br>**(1)**____________ **(2)**____________<br>**(3)**____________ **(4)**____________<br>**(5)**____________ **(6)** Other, which one? ____________<br>**(98)** DK/NA	__ __
**5.10.2 Did you have to complete any procedures <u>to get any of the tests authorized</u>?**<br>**(1)** Yes **(2)** No **(98)** DK/NA	
**5.10.3 Did you pay for the tests?**<br>**(1)** Yes **(2)** No→***q.5.11*** **(98)** DK/NA→***q.5.11***	__ __
**5.10.3.1 How much were you charged for the tests?**<br>____________________________________$<br>→ *If he/she does not remember how much, mark* **(99)**	__ __ __
**5.10.3.2 Why did you have to pay?**<br>____<br>____<br>____	__ __<br>__ __<br>__ __
**5.11 Did you have any <u>difficulty undergoing any of these ordered</u> by the emergency doctors?**<br>**(1)** Yes **(2)** No→***q.5.12*** **(98)** DK/NA→***q.5.12***	__ __
**5.11.1 What difficulties?**<br>____<br>____<br>____	__ __<br>__ __<br>__ __
**5.11.1.1 Do you think these difficulties affected your health?**<br>**(1)** Yes **(2)** No→***q.5.12*** **(98)** DK/NA→***q.5.12***	__ __

**5.11.1.2 How do you think they affected your health?**<br>____<br>____<br>____	___ ___<br>___ ___<br>___ ___
**5.12 Finally, can you tell me what the emergency doctor said you had?**<br>**(1)** Benign tumor →***q.5.13*** **(2)** Suspicious tumor →***q.5.13***<br>**(3)** Malignant tumor (cancer) **(4)** Other, which one? __________________ →***q.5.13***	___ ___
**5.12.1 Can you tell me when the emergency doctor <u>gave you this diagnosis</u>? (confirmed diagnosis)** → ***Write the answer*** *and continue with the questions if the date is not complete*<br>___ ___ / ___ ___/ ___ ___ ___ ___<br>D D / M M / Y Y Y Y<br>→ *Can you tell me the month and year?*<br>→ *If you he/she does not remember them, can you tell me how long it has been since <u>you were diagnosed</u>?* ____________________________________<br>→ *If he/she does not remember anything mark* **(99)**	___ ___ ___ ___<br>[*DAYS*]
**5.12.2 During this visit to the emergency department, did the doctor <u>refer you to a specialist for treatment recommendations</u>?**<br>**(1)** Yes→***q.5.14*** **(2)** No **(98)** DK/NA	___ ___
**5.13 Did the emergency doctor refer you for further investigation of your symptoms?**<br>**(1)** Yes **(2)** No →***Next section*** **(98)** DK/NA →***Next section***	___ ___
**5.13.1 Where did he/she refer you?**<br>**(1)** General doctor **(2)** Specialist **(3)** Hospital, which one? ______________________	___ ___
**5.14 In the emergency department, were you given a report for ______________________?**→ ***Fill in*** *according to the answer to question 5.13.1.*<br>**(1)** Yes **(2)** No **(98)** DK/NA →***Go to next section***	___ ___

6. EXPERIENCES IN HEALTH SERVICES FOR DIAGNOSIS: HOSPITALIZATION	
→ ***ASK*** *only if the hospitalization was in a hospital of the public health services of the network for cancer diagnosis as indicated in section 2 or question 5.8.*	
**6.1 Can you tell me when you were admitted to hospital?** → ***Write the answer*** *and continue with the questions if the date is not complete. If there are several entries, ask about the first one.*<br>___ ___ / ___ ___ / ___ ___ ___ ___<br>D D / M M / Y Y Y Y<br>→ *Can you tell me the month and year?*<br>→ *If he/she does not remember them, can you tell me how long it has been since <u>you were hospitalized</u>?*<br>______________________________<br>→ *If he/she does not remember anything mark* **(99)**	___ ___ ___ ___<br>*[DAYS]*
**6.2 On that occasion, do you remember how many days you were hospitalized?**<br>______________________________	___ ___ ___<br>*[DAYS]*
**6.3 <u>Did you have to complete any procedures to get</u> authorization for hospitalization?** (*Only Colombia*)<br>**(1)** Yes **(2)** No **(98)** DK/NA	___ ___
**6.4 <u>Did you pay for the hospitalization?</u>**<br>**(1)** Yes **(2)** No→***q.6.5*** **(98)** DK/NA→***q.6.5***	___ ___
**6.4.1 How much did you pay?**<br>______________________________$<br>→ *If he/she does not remember how much, mark* **(99)**	___ ___ ___
**6.4.2 Why did you have to pay?**<br>______________________________<br>______________________________<br>______________________________	___ ___<br>___ ___<br>___ ___
**6.5 How many times have you been hospitalized?** ______________________________	___ ___
**6.6 Finally, the doctor at the hospital, what did he/she said you had?** → ***Read*** *the response options*<br>**(1)** Benign tumor **(2)** Suspicious tumor<br>**(3)** Malignant tumor (cancer) **(4)** Other, which one?_____________<br>→ *If he/she answered options 1, 2, or 4, continue to the next section as indicated in 2.1.*	___ ___
**6.6.1 Can you tell me when you were informed of this diagnosis (confirmed diagnosis)?**<br>→ ***Write the answer*** *and continue with the questions if the date is not complete.*<br>___ ___ / ___ ___ / ___ ___ ___ ___<br>D D / M M / Y Y Y Y<br>→ *Can you tell me the month and year?*<br>→ *If he/she does not remember them, can you tell me how long it has been since <u>you received the diagnosis</u>?*<br>______________________________<br>→ *If he/she does not remember anything mark* **(99)**	___ ___ ___ ___<br>*[DAYS]*
**6.7 Did the doctor in charge of your hospital care <u>refer you to another specialist for treatment recommendation</u>?**<br>**(1)** Yes **(2)** No→***Next section*** **(98)** DK/NA→***Next section***	___ ___
**6.7.1 Can you tell me to which specialist you were referred to?** (*Add list of professionals by country*)<br>→ ***Read*** *the answer options according to cancer site.*<br>**(1)**_______________ **(2)**____________<br>**(3)**_______________ **(4)**____________<br>**(5)**_______________ **(98)** DK/NA	___ ___

7. DATA ON SOURCE OF CARE, MORBIDITY AND SOCIO-DEMOGRAPHIC CHARACTERISTICS

Read. *Next, I will ask you some general questions about social, economic and health issues.*

7.1 While you were diagnosed with cancer, have you suffered from any serious illnesses? → *If he/she does not remember them, read the list of diseases.*

(1) Lung disease (chronic obstructive pulmonary disease, pulmonary fibrosis or cystic fibrosis, asthma)
(2) Infarction (myocardial infarction)
(3) Heart Disease/Heart Failure (congestive)
(4) Disease of blood vessels/arteries and veins excluding those of the heart, brain and/or lungs (varicose veins, aneurysms, thromboses or embolisms)
(5) Stroke
(6) Dementia (Alzheimer's, other dementias)
(7) Anxiety
(8) Depression
(9) Rheumatologic/autoimmune diseases (lupus, rheumatoid arthritis, scleroderma, Sjogren's syndrome)
(10) Liver disease (cirrhosis, liver fibrosis)
(11) Diabetes
(12) Kidney disease (chronic renal failure, nephritic or nephrotic syndrome, single kidney patients)
(13) Obesity
(14) Hypertension
(15) HIV-AIDS
(16) COVID-19
(17) Other serious illness, which one? ____________
(97) None

___ ___
___ ___
___ ___

7.2 When you have a health problem, do you usually go to the same health center or hospital?
(1) Yes **(2)** No→***q.7.2.2*** **(98)** DK/NA→***q.7.2.2***

___ ___

7.2.1 Which health center or hospital?

____

____

___ ___
___ ___

7.2.2 What is your assigned health care center?

____

___ ___

7.3 Which ethnic group do you identify with? → ***Read*** *the response options* **(<u>*adapt according to country*</u>)**
(1) Mestizo **(2)** White **(3)** Mulatto **(4)** Black **(5)** Indigenous
(6) Other, which one? ________________________

___ ___

7.4 Age in completed years ________________________________

___ ___

7.5 Date of birth ___ ___ / ___ ___ / ___ ___ ___ ___
D D / M M / Y Y Y Y

___ ___

7.6 What is your country of birth?________________________________

___ ___

7.7 How long have you lived in this locality? (*Adapt according to country*)→ *If the person says 11 months or less enter 0 years.*
WRITE THE NUMBER OF YEARS ____________________

YEARS
___ ___

7.8 What is the highest level of education you have completed? (*Adapt according to country*) → ***Write the answer*** *and continue with the questions if the level of education is not specified.*

____

(1) None
(2) Incomplete primary education
(3) Complete primary education
(4) Incomplete secondary education
(5) Complete secondary education
(98) DK/NA
(6) Incomplete technical or technological education
(7) Complete technical or technological education
(8) Incomplete university education
(9) Complete university education
(10) Postgraduate education

___ ___

**7.9 What is your current employment situation?** →*If he/she is in more than one,* ***write down only the main one.*** *If he/she does not answer accurately, help to complete the answer.*<br>**(1)** Full-time employee<br>**(2)** Part-time employee<br>**(3)** Full-time self-employed person<br>**(4)** Part-time self-employed person<br>**(5)** Unemployed→***q.7.11***<br>**(6)** Student, school student, or in training→***q.7.11***<br>**(7)** Retired, early retirement or closed business→***q.7.11***<br>**(8)** Permanently unable to work→***q.7.11***<br>**(9)** Engaged in housework, children or others (paid)→***q.7.11***<br>**(10)** Engaged in housework, children or others (unpaid)→***q.7.11***<br>**(11)** Other kind of economic inactivity→***q.7.11***	___ ___
**7.10 What is your current occupation or main work activity?**<br>____	___ ___
**7.11 Which of the following options best describes your current living situation?** → ***Read*** *the response options*<br>**(1)** Living with a spouse or partner **(2)** Living alone →***q.7.16***<br>**(3)** Living with family/friends **(4)** Other, which one? ______________________<br>**(98)** DK/NA	___ ___
**7.12 Are you the head of the household?** → ***Concerning the person with the tumor.***<br>**(1)** Yes →***q.7.16*** **(2)** No	___ ___
**7.13 What is the highest level of education completed by the head of the household?** → ***Write the*** *answer and continue with the questions if the level of study is not specified.*<br>____<br>**(1)** None **(6)** Incomplete technical or technological education<br>**(2)** Incomplete primary education **(7)** Complete technical or technological education<br>**(3)** Complete primary education **(8)** Incomplete university education<br>**(4)** Incomplete secondary education **(9)** Complete university education<br>**(5)** Complete secondary education **(10)** Postgraduate education<br>**(98)** DK/NA	___ ___
**7.14 What is the current employment situation of the head of household?** → *If he/she is in more than one, write down only the main one. If he/she does not answer accurately, help to complete the answer.*<br>**(1)** Full-time employee<br>**(2)** Part-time employee<br>**(3)** Full-time self-employed person<br>**(4)** Part-time self-employed person<br>**(5)** Unemployed→***q.7.16***<br>**(6)** Student, school student, or in training →***q.7.16***<br>**(7)** Retired, early retirement or closed business→***q.7.16***<br>**(8)** Permanently unable to work→***q.7.16***<br>**(9)** Engaged in housework, children or others (paid)→***q.7.16***<br>**(10)** Engaged in housework, children or others (unpaid)→***q.7.16***<br>**(11)** Other kind of economic inactivity________________________→***q.7.16***	___ ___
**7.15 What is the current occupation of the head of the household?**<br>____	___ ___
**7.16 Do you have private health insurance?** (Adapt the section to each country including public insurance)<br>**(1)** Yes **(2)** No **(98)** DK/NA	___ ___

**7.17 How much money did all household members earn in total in the last month? → *Write down the first answer*** *and continue with the questions if the income amount is not clearly specified*<br>______________________________$<br>**(1)** No income **(6)** From $(*Complete*) to $(*Complete*) (3-4 minimum wages)<br>**(2)** Up to $(*Complete*) (1/2 minimum wage) **(7)** From $(*Complete*) to $(*Complete*) (4-5 minimum wages)<br>**(3)** From $(*Complete*) to $(*Complete*) (1/2-1 minimum wage) **(8)** From $(*Complete*) to $(*Complete*) (5-10 minimum wages)<br>**(4)** From $(*Complete*) to $(*Complete*) (1-2 minimum wages) **(9)** From $(*Complete*) to $(*Complete*) (10-15 minimum wages)<br>**(5)** From $$(*Complete*) to $(*Complete*) (2-3 minimum wages) **(10)** More than $(*Complete*) (more than 15 minimum wages)	___ ___
→ If he/she answered that "live alone" on q.7.11, go to q.20<br>**7.18 ¿ How many people usually make up the household, including yourself (persons sharing food and living expenses)?**<br>*WRITE THE NUMBER OF PERSONS* ______________________	___ ___
**7.19 How many rooms do people sleep in?**<br>*WRITE THE NUMBER OF ROOMS* ______________________	___ ___
**7.20 Register the sex**<br>**(1)** Man **(2)** Woman	___ ___
→Ask this question in cases where the respondent is a caregiver or family member	
**7.21 What is your relationship to the patient?**<br>**(1)** Son/Daughter **(2)** Mother/Father **(3)** Grandson/Granddaughter **(4)** Grandparent **(5)** Partner **(6)** Paid caregiver **(7)** Other, who?______________	___ ___
**7.22 Are there any additional comments you would like to make (respondent/caregiver or family member)?**<br>______________________<br>______________________<br>______________________	___ ___

8. FILL IN AFTER THE SURVEY	
→ ***Mention*** *to the person that the interview may need to be repeated to ensure the quality of the data**.***	
**Would you be interested in receiving the results of the study?**<br>**(1)** Yes→ *PLEASE NOTE CONTACT DATA (email, phone) IN INFORMED CONSENT*<br>**(2)** No	___ ___
**Would you be willing to participate in an interview to share more about your experience during cancer diagnosis?**<br>**(1)** Yes→ *RECORD THE CONTACT INFORMATION (email, phone number) IN CONSENT FORM*<br>**(2)** No	___ ___
→*To be filled in by the interviewer*	
**8.1 Name of person answering the questionnaire** → ***Exclude surnames***<br>____	___ ___
**8.2 How would you rate the cooperation of the interviewee?**<br>**(1)** Good **(2)** Fair **(3)** Poor	___ ___
**8.3 How well did the interviewee understand the questions?**<br>**(1)** Good **(2)** Fair **(3)** Poor	___ ___
**8.4 Time of completion ___ ___ : ___ ___**	___ ___
**8.5 Interview location**<br>**(1)** In-person at home<br>**(2)** In-person at a health facility<br>**(3)** Remote online<br>**(4)** Remote by telephone	___ ___
**8.6 Use of patient documents to complete information on dates and tests**<br>**(1)** Yes **(2)** No	
**General observations by the interviewer:**<br>____<br>____<br>____	___ ___<br>___ ___<br>___ ___
