# Supplementary material for: Delays and detours during cancer diagnosis: a cross-sectional study on patient pathways and provider intervals in public healthcare networks of Chile, Colombia and Ecuador
Source: BMC Cancer. 2026 Feb 26;26:438. doi: 10.1186/s12885-026-15737-5 (PMC13059593; doi:10.1186/s12885-026-15737-5)
Supplement: Supplementary file 2 — Additional file 2. Table 1S. Flow of patient identification, responses and rejection in Chile, Colombia and Ecuador. Table 2S. Diagnostic intervals (in days) to cancer diagnosis for all patients, and according to symptomatic and non-symptomatic in the studied public healthcare networks of Chile, Colombia, and Ecuador. Table 3S. Diagnostic intervals (in days) to cancer diagnosis according to most frequent cancer site in the studied public healthcare networks of Chile, Colombia, and Ecuador. Table 4S. Patient pathways to cancer diagnosis for symptomatic and non-symptomatic patients according to type of pathway (public or private) in Chile, Colombia, and Ecuador. Table 5S. Number of contacts to health services (visits, hospitalizations and tests) until cancer diagnosis according to patient pathways in Chile, Colombia and Ecuador. Table 6S. Characteristics of patients with a provider interval longer than 365 days in the studied networks of Chile, Colombia and Ecuador.in Latin America. [file 12885_2026_15737_MOESM2_ESM.docx]

***Additional material***

**Table 1S.** Flow of patient identification, responses and rejection in Chile, Colombia and Ecuador

|  | **Chile**  **n(%)** | **Colombia**  **n(%)** | **Ecuador n(%)** | **Total n(%)** |
| --- | --- | --- | --- | --- |
| **Identified and contacted** | 643 (100) | 5.709 (100) | 1.965 (100) | 8.317 (100) |
| **Respondents *(% of total***  ***contacted patients)*** | 635 (98.8) | 3.965 (69,5) | 1.411 (71,8) | 6.013 (72.3) |
| Did not fulfill inclusion criteria | 137 (21.6) | 2.561(64.6) | 908 (64.4) | 3.606 (60.0) |
| Patient died | 54 (8.5) | 772 (19.5) | 71 (5.0) | 897 (14.2) |
| Not willing to participate^1^ | 93 (14.6) | 284 (7.2) | 67 (4.7) | 444 (10.2) |
| Lack of interest | 51 (54.8) | 222 (78.2) | 22 (32.8) | 295 (66.4) |
| Poor health | 21 (22.6) | 25 (8.8) | 23 (34.3) | 69 (15.5) |
| Lack of time | 21 (22.6) | 37 (13.0) | 22 (32.8) | 80 (18.0) |
| **Participants^2^** | 351 (79.1) | 348 (55.71) | 365 (84.5) | 1064 (70.5) |

^1^In some cases, it was not possible to verify if they fulfilled the inclusion criteria.

^2^Percentage calculated based on the total number of respondents who fulfilled the inclusion criteria and were alive.

**Table 2S.** Diagnostic intervals (in days) to cancer diagnosis for all patients, and according to symptomatic and non-symptomatic in the studied public healthcare networks of Chile, Colombia, and Ecuador.

|  | **Chile** | **Colombia** | **Ecuador** |
| --- | --- | --- | --- |
| **All patients** | **N=351** | **N=303** | **N= 365** |
| **Total diagnostic interval** | **(n=350)** | **(n=292)** | **(n=365)** |
| Percentile 50 | 129 | 215 | 151 |
| Percentile 75 | 273 | 414 | 320 |
| Percentile 90 | 558 | 744 | 558 |
| IQR | 198 | 313 | 259 |
| *Missing* | *1* | *11* | 53 |
| **Provider interval** | **(n=350)** | **(n=292)** | **(n=365)** |
| Percentile 50 | 111 | 156 | 92 |
| Percentile 75 | 195 | 339 | 212 |
| Percentile 90 | 425 | 670 | 454 |
| IQR | 134 | 275 | 173 |
| *Missing* | *1* | *11* | 8 |
| **Symptomatic patients** | ***n=209*** | ***n=262*** | ***n=308*** |
| **Total diagnostic interval** | **(n=208)** | **(n=255)** | **(n=308)** |
| Percentile 50 | 147 | 221 | 151 |
| Percentile 75 | 299 | 425 | 317 |
| Percentile 90 | 710 | 801 | 553 |
| IQR | 213 | 324 | 254 |
| *Missing* | *1* | *7* | *48* |
| **Patient interval** | **(n=209)** | **(n= 251)** | **(n=308)** |
| Percentile 50 | 11 | 15 | 12 |
| Percentile 75 | 59 | 62 | 61 |
| Percentile 90 | 212 | 156 | 200 |
| IQR | 57 | 62 | 61 |
| *Missing* | *0* | *11* | *66* |
| **Provider interval** | **(n=208)** | **(n=255)** | **(n=308)** |
| Percentile 50 | 111 | 154 | 92 |
| Percentile 75 | 206 | 334 | 212 |
| Percentile 90 | 461 | 674 | 454 |
| IQR | 149 | 273 | 174 |
| *Missing* | *1* | *7* | *8* |
| **Non-symptomatic patients** | ***n=142*** | ***n=41*** | **n=57** |
| **Total diagnostic interval** | **(n=142)** | **(n=37)** | **(n=57)** |
| Percentile 50 | 114 | 191 | 142 |
| Percentile 75 | 191 | 365 | 377 |
| Percentile 90 | 351 | 625 | 689 |
| IQR | 130 | 268 | 318 |
| *Missing* | *0* | *4* | 5 |
| **Provider interval** | **(n=142)** | **(n=37)** | **(n=57)** |
| Percentile 50 | 114 | 191 | 92 |
| Percentile 75 | 191 | 365 | 245 |
| Percentile 90 | 351 | 625 | 487 |
| IQR | 130 | 268 | 200 |
| *Missing* | *0* | *4* | 0 |

**Total diagnostic interval**: time elapsed from the date of identification of the problem (through symptoms discovery or an abnormal result from an opportunistic screening or casual finding) up to diagnostic confirmation. **Patient interval**: time elapsed between the date of perception of symptoms and first appointment request to a health service/emergency visit. **Provider interval**: time elapsed between the date of the first appointment request to a health service/emergency visit for the symptoms (or opportunistic screening / casual finding), and the diagnostic confirmation.

**Table 3S.** Diagnostic intervals (in days) to cancer diagnosis according to most frequent cancer site in the studied public healthcare networks of Chile, Colombia, and Ecuador.

|  | **Chile** | **Colombia** | **Ecuador** |
| --- | --- | --- | --- |
| **Breast** | ***n=131*** | ***n=51*** | **n=177** |
| **Total diagnostic interval** | **(n=131)** | **(n=49)** | **(n=177)** |
| Percentile 50 | 111 | 235 | 182 |
| Percentile 75 | 160 | 371 | 346 |
| Percentile 90 | 286 | 691 | 557 |
| IQR | 88 | 251 | 285 |
| *Missing* | *0* | *2* | 22 |
| **Patient interval** | **(n=65)** | **(n=40)** | **(n=157)** |
| Percentile 50 | 11 | 15 | 30 |
| Percentile 75 | 40 | 62 | 76 |
| Percentile 90 | 169 | 118 | 243 |
| IQR | 48 | 62 | 76 |
| *Missing* | *0* | *1* | 28 |
| **Provider interval** | **(n=131)** | **(n=49)** | **(n=177)** |
| Percentile 50 | 94 | 212 | 104 |
| Percentile 75 | 136 | 327 | 235 |
| Percentile 90 | 203 | 691 | 440 |
| IQR | 76 | 243 | 189 |
| *Missing* | 0 | *2* | 3 |
| **Colorectal** | ***n=89*** | ***n=49*** | **n=54** |
| **Total diagnostic interval** | **(n=88)** | **(n=48)** | **(n=54)** |
| Percentile 50 | 118 | 181 | 115 |
| Percentile 75 | 252 | 347 | 332 |
| Percentile 90 | 851 | 497 | 639 |
| IQR | 190 | 273 | 288 |
| *Missing* | *1* | *1* | *8* |
| **Patient interval** | **(n=70)** | **(n=46)** | **(n=54)** |
| Percentile 50 | 11 | 11 | 3 |
| Percentile 75 | 59 | 31 | 61 |
| Percentile 90 | 344 | 92 | 212 |
| IQR | 57 | 31 | 61 |
| *Missing* | *0* | *3* | *10* |
| **Provider interval** | **(n=88)** | **(n=47)** | **(n=54)** |
| Percentile 50 | 104 | 118 | 72 |
| Percentile 75 | 168 | 271 | 153 |
| Percentile 90 | 426 | 449 | 442 |
| IQR | 128 | 222 | 125 |
| *Missing* | 0 | *2* | *1* |
| **Prostate** | ***n= 61*** | ***n=27*** | **n=28** |
| **Total diagnostic interval** | **(n=61)** | **(n=26)** | **(n=28)** |
| Percentile 50 | 273 | 219.5 | 132 |
| Percentile 75 | 403 | 369 | 221 |
| Percentile 90 | 700 | 1115 | 487 |
| IQR | 280 | 228 | 159 |
| *Missing* | *0* | *1* | 5 |
| **Patient interval** | **(n=25)** | **(n=18)** | **(n=19)** |
| Percentile 50 | 11 | 61 | 31 |
| Percentile 75 | 30 | 90 | 89 |
| Percentile 90 | 120 | 180 | 152 |
| IQR | 30 | 88 | 90 |
| *Missing* | *0* | *1* | 5 |
| **Provider interval** | **(n=61)** | **(n=27)** | **(n=28)** |
| Percentile 50 | 224 | 169 | 71 |
| Percentile 75 | 364 | 295 | 199 |
| Percentile 90 | 700 | 740 | 330 |
| IQR | 226 | 234 | 170 |
| *Missing* | 0 | 0 | 0 |
| **Stomach** | ***n=19*** | ***n=41*** | **n=43** |
| **Total diagnostic interval** | **(n=19)** | **(n=39)** | **(n=43)** |
| Percentile 50 | 99 | 245 | 137 |
| Percentile 75 | 253 | 382 | 243 |
| Percentile 90 | 426 | 611 | 474 |
| IQR | 205 | 285 | 181 |
| *Missing* | *0* | *2* | 8 |
| **Patient interval** | **(n=17)** | **(n=36)** | **(n=39)** |
| Percentile 50 | 10 | 25 | 0 |
| Percentile 75 | 51 | 85 | 61 |
| Percentile 90 | 212 | 304 | 90 |
| IQR | 54 | 85 | 61 |
| *Missing* | *0* | *2* | *12* |
| **Provider interval** | **(n=19)** | **(n=38)** | **(n=43)** |
| Percentile 50 | 57 | 137 | 85 |
| Percentile 75 | 92 | 296 | 188 |
| Percentile 90 | 425 | 531 | 352 |
| IQR | 52 | 245 | 148 |
| *Missing* | *0* | *3* | *3* |
| **Cervix** | ***NA*** | ***n=53*** | **n=63** |
| **Total diagnostic interval** |  | **(n=50)** | **(n=63)** |
| Percentile 50 |  | 270.5 | 126 |
| Percentile 75 |  | 428 | 395 |
| Percentile 90 |  | 1045 | 822 |
| IQR |  | 314 | 334 |
| *Missing* |  | *3* | 10 |
| **Patient interval** |  | **(n=39)** | **(n=45)** |
| Percentile 50 |  | 11 | 0 |
| Percentile 75 |  | 62 | 20 |
| Percentile 90 |  | 198 | 59 |
| IQR |  | 62 | 20 |
| *Missing* |  | *2* | *11* |
| **Provider interval** |  | **(n=51)** | **(n=63)** |
| Percentile 50 |  | 183 | 92 |
| Percentile 75 |  | 407 | 227 |
| Percentile 90 |  | 1008 | 668 |
| IQR |  | 329 | 177 |
| *Missing* |  | *2* | 1 |

**Total diagnostic interval**: time elapsed from the date of identification of the problem (through symptoms discovery or an abnormal result from an opportunistic screening or casual finding) up to diagnostic confirmation. **Patient interval**: time elapsed between the date of perception of symptoms and first appointment request to a health service/emergency visit. **Provider interval**: time elapsed between the date of the first appointment request to a health service/emergency visit for the symptoms (or opportunistic screening / casual finding), and the diagnostic confirmation.

***NA:*** Not applicable, as selected cancer were different among countries.

**Table 4S.** Patient pathways to cancer diagnosis for symptomatic and non-symptomatic patients according to type of pathway (public or private) in Chile, Colombia, and Ecuador.

|  | **Chile**  **N=351** | | **Colombia**  **N=303** | | **Ecuador**  **N=365** | |
| --- | --- | --- | --- | --- | --- | --- |
|  | ***Symptomatic*** | ***Non-symptomatic*** | ***Symptomatic*** | ***Non-symptomatic*** | ***Symptomatic*** | ***Non-symptomatic*** |
|  | *n (%) n (%)* | | *n (%)* | *n (%)* | *n (%)* | *n (%)* |
| **Diagnostic pathways only in public services** | **35 (16.7) ^1^** | **46 (32.4)** | **170 (64.9) ^1^** | **34 (82.9)** | **122 (39.6)** | **27 (47.4)** |
| **Entry by PC, referred to SC(OC) and diagnosed after few visits in SC (<3) services** | **23 (11)** | **33 (23.2)** | **45 (17.2)** | **9 (22.0)** | **52 (16.9)** | **15 (26.3)** |
| **Entry by ES and use only of specialized services until diagnosis** | **7 (3.3)** | **6 (4.2)** | **54 (20.6)** | **6 (14.6)** | **38 (12.3)** | **2 (3.5)** |
| **Entry by SC(OC) and diagnosed after use of SC services (OC, hospitalization, ES)** | **1 (0.5)** | **5 (3.5)** | **7 (2.7)** | **13 (31.7)** | **23 (7.5)** | **7 (12.3)** |
| **Other pathways involving going back and forth between services and/or levels of care** | **4 (1.9)** | **2 (1.4)** | **64(24.4)** | **6 (14.6)** | **9 (2.9)** | **3 (5.3)** |
| Entry by PC and use mostly of ES and hospitalization | 1 (0.5) | 1 (0.7) | 22 (8.4) | 1 (2.4) | 3 (1.0) | 0 (0) |
| Entry by PC and back and forth between care levels | 3 (1.4) | 1 (0.7) | 24 (9.1) | 3 (7.3) | 3 (1.0) | 2 (3.5) |
| Entry by PC, after few visits (PC**≤3**) referred to SC(OC), and use of different specialized services (OC, hospitalization, ES) | - | - | 18 (6.9) | 2 (4.9) | 3 (1.0) | 1 (1.8) |
| **Diagnostic pathways in public and private services** | **174 (83.3) ^1^** | **96 (67.6)** | **92 (35.1) ^1^** | **7 (17.1)** | **186 (60.4)** | **30 (52.6)** |
| **Public pathways complementing with some private services** | **74 (35.4)** | **34 (23.9)** | **85 (32.4)** | **7 (17.1)** | **163 (52.9)** | **29 (50.9)** |
| **Private pathways ending in public services** | **100 (47.8)** | **62 (43.7)** | **7 (2.7)** | **0 (0.0)** | **23 (7.5)** | **1 (1.8)** |

**PC:** Public primary care; **SC (OC):** Public Secondary care Outpatient Consultation; **ES:** Emergency Services.

**For Ecuador**, health services of the complementary health network (IESS, ISSFA o ISSPOL) are included in the public healthcare services categories.

**^1^** Statistically significant difference between symptomatic and asymptomatic when comparing diagnostic pathway public-only and public-private pathway, using chi-square test (p<0.01).

|  | **Chile N=351** | | **Colombia N=303** | | **Ecuador N=365** | |
| --- | --- | --- | --- | --- | --- | --- |
|  | **n** | *Median (IQR)* | **n** | *Median (IQR)* | **n** | *Median (IQR)* |
| **In patient pathways only in public services (*)** | **81** | **3 (2-5)** | **204** | **5 (3-7)** | **149** | **3 (2-4)** |
| Entry by PC, referred to SC(OC) and diagnosed after few visits in SC (<3) services | 56 | 3 (2-4) | 54 | 4 (3-5) | 67 | 4 (3-5) |
| Entry by ES and use only of specialized services until diagnosis | 13 | 4 (2-6) | 60 | 4 (3-6) | 40 | 2 (1-3) |
| Entry by SC(OC) and diagnosed after use of few (≤5) SC(OC) services, hospitalization, and ES | 6 | 2 (2-4) | 20 | 3 (2-4) | 30 | 3 (2-4) |
| Other pathways involving going back and forth between services and/or levels of care | 6 | 5 (3-5) | 70 | 7 (5-8) | 12 | 5 (2-6) |
| **In patient pathways in public and private services** | **270** | **7 (6-9)** | **99** | **7 (5-9)** | **216** | **4 (3-5)** |
| Public pathways complementing with some private services | 108 | 6 (5-9) | 92 | 7 (5-9) | 192 | 4 (3-5) |
| Private pathways ending in public services | 162 | 7 (6-9) | 7 | 7 (5-8) | 24 | 2 (2-3) |

**Table 5S** Number of contacts to health services (visits, hospitalizations and tests) until cancer diagnosis according to patient pathways in Chile, Colombia and Ecuador

(*) In public diagnostic pathways, and in Ecuador also in the public-private pathways, diagnostic tests prescribed by the doctor are considered in the same episode

and are counted within the medical visit.

**Table 6S.** Characteristics of patients with a provider interval longer than 365 days in the studied networks of Chile, Colombia and Ecuador.

| **Variables** | **Chile**  **n= 42** | **Colombia**  **n=64** | **Ecuador**  **n= 51** |
| --- | --- | --- | --- |
|  | *n(%)* | *n(%)* | *n(%)* |
| **Sex** |  |  |  |
| Woman | 20 (47.6) | 45 (70.3) | 46 (90.2) |
| Man | 22 (52.4) | 19 (29.7) | 5 (9.8) |
| **Age** |  |  |  |
| 18-39 | 0 (0.0) | 5 (7.8) | 12 (23.5) |
| 40-59 | 11 (26.2) | 24 (37.5) | 23 (45.1) |
| 60-79 | 27 (64.3) | 31 (48.4) | 13 (25.5) |
| 80 or more | 4 (9.5) | 4 (6.3) | 1 (2.0) |
| **Level of education** |  |  |  |
| No studies or incomplete primary | 17 (40.5) | 27 (42.2) | 5 (9.8) |
| Complete primary | 7 (16.6) | 22 (34.4) | 8 (15.7) |
| Secondary and further | 18 (32.8) | 15 (23.4) | 38 (74.5) |
| **Cancer site** |  |  |  |
| Breast | 5 (11.9) | 11 (17.2) | 23 (45.1) |
| Colorectal | 11 (26.2) | 8 (12.5) | 9 (17.6) |
| Prostate | 15 (35.7) | 5 (7.8) | 2 (3.9) |
| Lung | 4 (9.5) | 1 (1.6) | NA |
| Urinary tract (kidney and bladder) | 5 (11.9) | 1 (1.6) | NA |
| Stomach | 2 (4.8) | 7 (10.9) | 4 (7.8) |
| Cervix | *NA* | 14 (21.9) | 13 (25.5) |
| Melanoma | *NA* | 1 (1.6) | NA |
| Other | *NA* | 16 (25.0) | NA |
| **Cancer stage** |  |  |  |
| I | 12 (28.6) | *n/a* | 2 (3.9) |
| II | 12 (28.6) | *n/a* | 13 (25.5) |
| III | 10 (23.8) | *n/a* | 15 (29.4) |
| IV | 7 (16.7) | *n/a* | 4 (7.8) |
| *Missing* | *1 (2.4)* | *n/a* | 17 (33.33) |
| **Identification of the health problem** |  |  |  |
| Symptomatic | 29 (69) | 55 (85.9) | 41 (80.4) |
| Non symptomatic | 13 (31) | 9 (14.1) | 10 (19.6) |
| **Type of diagnostic pathway** |  |  |  |
| **Pathways only in public services** | **7 (16.7)** | **41 (64.1)** | **21 (41.2)** |
| Entry by PC, referred to SC(OC) | 7 (16.7) | 13 (20.3) | 12 (23.5) |
| Entry by ES and use only of specialized services until diagnosis |  | 2 (3.1) | 2 (3.9) |
| Entry by SC(OC) and diagnosed after use of SC services |  | 3 (4.7) | 3 (5.9) |
| Other pathways |  | 23 (35.9) | 4 (7.8) |
| **Pathways in public and private services** | **35 (83.3)** | **23 (35.9)** | **30 (58.8)** |
| Public pathways complementing with some private services | 23 (54.7) | 23 (35.9) | 29 (56.9) |
| Private pathways ending in public services | 12 (28.6) | 0 (0.0) | 1 (2.0) |

**Category other type of cancer in Colombia:** thyroid, ovary, brain, vagina, pancreas, soft tissue, tongue, larynx, eye, salivary/parotid.

***NA:*** Not applicable, as selected cancer were different among countries.

***n/a*:** Data on cancer stage is not available for Colombia.

**Data source of cancer stage:** Clinical registers in Chile and Ecuador.
